# Age- and Sex-Specific Prevalence and Modifiable Risk Factors of Mild Cognitive Impairment Among Older Adults in China: A Population-Based Observational Study

**DOI:** 10.3389/fnagi.2020.578742

**Published:** 2020-10-30

**Authors:** Jingzhu Fu, Qian Liu, Yue Du, Yun Zhu, Changqing Sun, Hongyan Lin, Mengdi Jin, Fei Ma, Wen Li, Huan Liu, Xumei Zhang, Yongjie Chen, Zhuoyu Sun, Guangshun Wang, Guowei Huang

**Affiliations:** ^1^Department of Nutrition and Food Science, School of Public Health, Tianjin Medical University, Tianjin, China; ^2^Tianjin Key Laboratory of Environment, Nutrition and Public Health, Tianjin, China; ^3^Department of Social Medicine and Health Management, School of Public Health, Tianjin Medical University, Tianjin, China; ^4^Department of Epidemiology and Biostatistics, School of Public Health, Tianjin Medical University, Tianjin, China; ^5^Neurosurgical Department of Baodi Clinical College of Tianjin Medical University, Tianjin, China; ^6^Department of Tumor, Baodi Clinical College of Tianjin Medical University, Tianjin, China

**Keywords:** mild cognitive impairment, prevalence, risk factors, sex differences, age differences, older adults

## Abstract

**Background:**

Minimal data are available on the prevalence of mild cognitive impairment (MCI) in older Chinese adults. Moreover, the current information on MCI shows important geographical variations.

**Objective:**

We aimed to assess the prevalence and risk factors for MCI by age and sex among older adults in a North Chinese population.

**Methods:**

In this population-based cross-sectional study, we enrolled a random sample of 4,943 adults aged ≥ 60 years between March 2018 and June 2019 in Tianjin, China. Of these, 312 individuals were excluded due to a lack of data (e.g., fasting blood test). As a result, 4,631 subjects were assessed. Individuals with MCI were identified using neuropsychological assessments, including the Mini-Mental State Examination and Activities of Daily Living scale, based on a modified version of the Petersen’s criteria.

**Results:**

The mean (SD) age of the 4,631 participants was 67.6 (4.89) years, and 2,579 (55.7%) were female. The overall age- and sex-standardized prevalence of MCI in our study population was 10.7%. There were significant associations of MCI with age [65–69 vs. 60–64 years, OR = 0.74; 95% confidence interval (CI): 0.58, 0.96], physical activity (≥23.0 vs. <23.0 MET-hours/week, OR = 0.79; 95% CI: 0.64, 0.96), body mass index (BMI) (OR = 0.92; 95% CI: 0.89, 0.95), grip strength (OR = 0.50; 95% CI: 0.38, 0.67), hypertension (yes vs. no, OR = 1.44; 95% CI: 1.18, 1.77), higher levels of sleepiness (OR = 1.80; 95% CI: 1.36, 2.37), and longer sleep duration (OR = 1.40; 95% CI: 1.14, 1.72). The inverse association between BMI and MCI was stronger in older age groups (*P* for heterogeneity = 0.003). Moreover, the magnitude of association between triglycerides and MCI was different between the sexes (*P* for heterogeneity = 0.029).

**Conclusion:**

The age- and sex-standardized prevalence of MCI was 10.7% in the study sample. Physical activity, BMI, grip strength, sleepiness, sleep duration, and hypertension were associated with the prevalence of MCI. Additionally, triglycerides and BMI might be differently associated with the presence of MCI for different sexes and age stages, respectively.

## Introduction

Dementia is a common geriatric illness that is characterized by a decline in cognition that inhibits daily function and places a significant burden on patients, families, and social care systems (Langa, 2018, Cognitive Aging). Mild cognitive impairment (MCI) is a transitional state between normal aging and dementia, and approximately 10–20% of MCI patients progress annually to dementia (Winblad et al., 2004; Subramanyam and Singh, 2016). While there is no effective treatment available for the MCI-to-dementia progression, the burden of the disease can be reduced through primary prevention.

In recent decades, the rapid growth of the elderly population in China has spurred research interest in the cause and prevention of MCI. Previous epidemiological studies have demonstrated that sociodemographic, lifestyle, and vascular factors may be associated with MCI risk (Chiam et al., 2004; Lee et al., 2009; Qiu et al., 2010; Mohan et al., 2019). Since aging and certain hormonal changes (e.g., estrogen) involve a heightened susceptibility to cognitive decline, the association between lifestyle and MCI may vary depending on age and sex. However, few studies have examined the age- and sex-related differences in risk factors for MCI (House et al., 1988; Williams and Umberson, 2004). Our literature review only identified one cross-sectional study from China that analyzed the association between modifiable risk factors and MCI stratified by sex (Zhang et al., 2019). However, that study had a small sample size, lacked a formal test for heterogeneity, and only examined a limited number of predictors. Unlike other countries where no sex differences in the prevalence of MCI have been observed (Au et al., 2017), data from China have shown that the prevalence of MCI is higher in females than males (Nie et al., 2011). Additionally, a meta-analysis revealed that there was a difference in the prevalence of MCI between North and South China, but detailed data regarding the prevalence of MCI in Northern China have remained sparse (Nie et al., 2011).

In this study, we investigated the sex and age differences in the prevalence of MCI and the association between multiple influencing factors and MCI stratified by age and sex in a large-scale cross-sectional study in Northern China. This study should help to gain a better understanding of MCI and strategies to protect older people against cognitive decline.

## Materials and Methods

### Study Population

**FIGURE 1:**
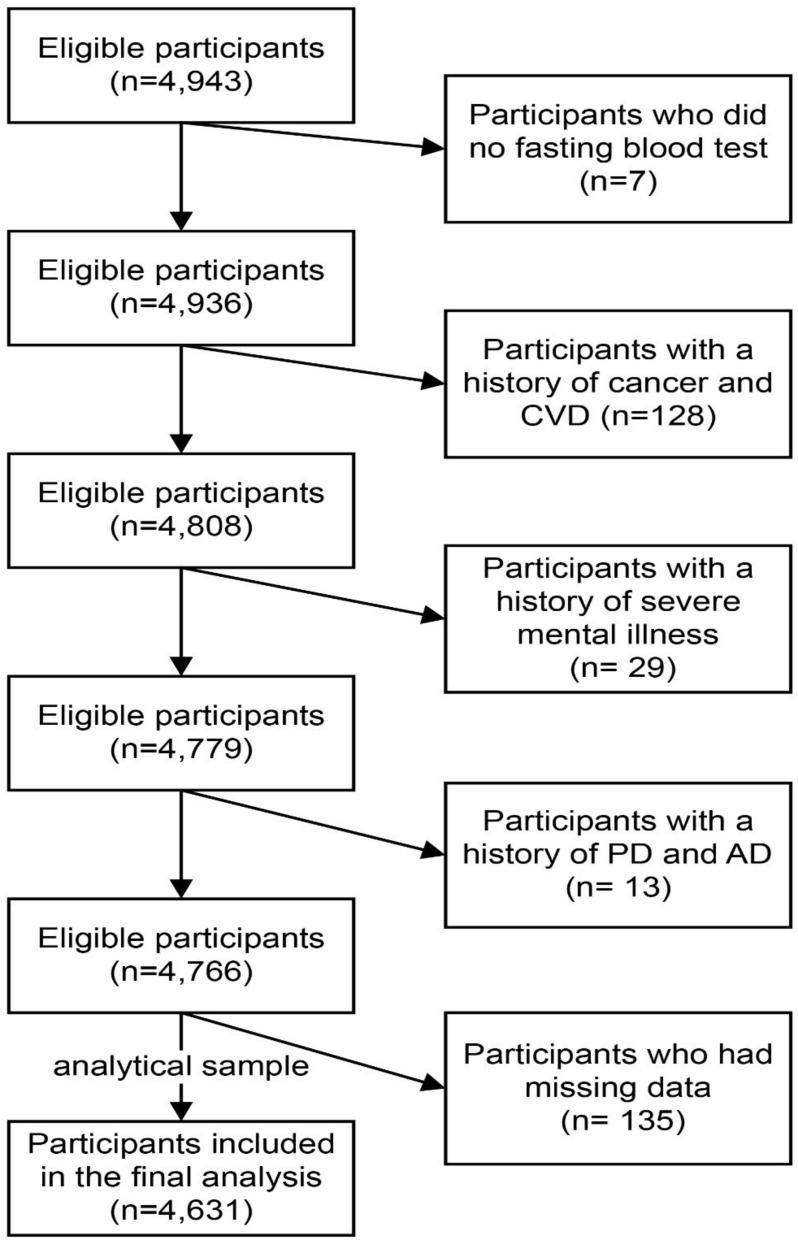
Flow diagram showing the selection of the study population. CVD, cerebrovascular disease; PD, Parkinson’s disease; AD, Alzheimer’s disease.

This cross-sectional analysis used data collected at baseline from the Tianjin Elderly Nutrition and Cognition Cohort study (Clinical Trials Registration Identifier: ChiCTR2000034348), an ongoing elderly population-based prospective cohort study focused on the relationship between nutrition and cognition in China. Briefly, participants were recruited from the Baodi area of Tianjin, China. All participants had sufficient mobility, vision, and hearing to complete the assessments, and were aged 60 years or older at enrollment between March 2018 and June 2019. Using multistage cluster sampling, we randomly selected three communities in the Baodi District. From the three communities, we identified a total of 5,577 eligible subjects. Those who were willing to participate underwent a thorough clinical examination, personal interview, and cognitive function assessment, administered by licensed physicians, trained graduate students, and psychologists, respectively (*n* = 4,943; participation rate = 88.6%). Subjects who did not undergo a fasting blood test (*n* = 7), had a history of cerebrovascular disease (*n* = 91), cancer (*n* = 37), severe mental illness (*n* = 29), Parkinson’s disease (*n* = 9), and Alzheimer’s disease (AD) (*n* = 4) were excluded. As such, 4,766 subjects were included in the dataset (mean [standard deviation (SD)] age: 67.6 (4.89) years; males, 44.3%) (Figure 1). The study protocols were approved by the Ethics Committee of Tianjin Medical University, China (approval/protocol number: TMUhMEC2018013), and all participants provided their written informed consent before participating in the study. If a participant was illiterate, then informed consent was sought from their legal representative. All experimental procedures adhered strictly to the study protocol.

### Definition of Mild Cognitive Impairment

We used a modified version of the Petersen’s criteria (Petersen, 2004) to diagnose MCI: (1) subjective memory complaints over at least 6 months, preferably corroborated by an informant; (2) a Mini-Mental State Examination (MMSE) score of ≤17 points for illiterate participants, ≤20 points for those with primary school education, and ≤24 points for those with secondary education or above (Katzman et al., 1988); (3) absence of dementia (Diagnostic and Statistical Manual of Mental Disorders-IV criteria), AD (National Institute of Neurological and Communicative Disorders and Stroke, and the Alzheimer’s Disease and Related Disorders Association criteria), psychiatric disorders, cerebral damage, or physical diseases leading to cognitive impairment; (4) cognitive performance indicated by 1.5 SD below the age-corrected (and education, where available) norms on at least one test in the neuropsychological battery (Ritchie et al., 2001); and (5) no/minimal impairment of daily life activities, as measured by the Activities of Daily Living Scale (ADL) (<26 points) (Perneczky et al., 2006). MCI patients had to fulfill the above five criteria, and the diagnosis of MCI was based on an expert consensus by a panel of physicians, neurologists, neuropsychologists, and psychiatrists.

### Measures

We focused specifically on potential risk factors for MCI, including sociodemographic (e.g., sex and age) and health-related variables (e.g., lifestyle and physical performance). These variables were collected via a face-to-face interview or clinical examination according to a structured protocol.

The sociodemographic characteristics included age (60–64, 65–69, 70–74, and ≥75 years), sex, education level (illiterate, primary school, or middle school; high school, and above), household income (<3,000, 3,000–5,000, and >5,000 RMB), employment status (working full or part-time; not working or retired), marital status (married; single, divorced, or widowed).

Lifestyle variables included smoking, alcohol drinking, physical activity, and sleep characteristics. Smoking status was grouped by current smoker, ex-smoker, or never smoked. Drinking status was grouped as current drinker or non-drinker. Physical activity (PA) was measured using a short version of the International Physical Activity Questionnaire, which collects information on the number of minutes spent on vigorous-intensity activities, moderate-intensity activities, walking, and sitting during the past week (Craig et al., 2003). Total PA, expressed in metabolic equivalent hours per week (MET-h/week), was calculated by multiplying the hours per week of vigorous, moderate, and walking activities with their corresponding MET coefficients (8.0, 4.0, and 3.3, respectively) and then summing the scores (Craig et al., 2003). The level of total PA was divided into two categories: <23 and ≥23 METs-h/week (Cao, 2015). Self-reported sleep characteristics were derived from participants’ sleep duration and daytime sleepiness. Sleep duration was assessed by asking the question: “How many hours do you usually sleep at night?” Self-reported sleep duration was examined in categories of short sleep duration (<6.5 h) and long sleep duration (>8.5 h), with 6.5–8.5 h of sleep as the reference (Keage et al., 2012; Chiu et al., 2016). Besides, the eight-item Epworth Sleepiness Scale was used to assess the likelihood of falling asleep in common daily situations (Johns, 1992). Each item scored on a four-point scale was summed with scores ranging from 0 to 24, with higher scores indicating greater sleepiness. The scale has well-established validity and reliability (Johns, 1992).

Physical performance was assessed by grip strength (GS). GS was measured using an electronic handheld dynamometer (EH101; CAMRY, Guangdong, China). Participants were tested by trained technicians under the same conditions. Forces were measured twice for each hand, and the greatest force was used for the analyses. Additionally, GS relative to body weight (kg/kg) was also calculated because of the involvement of body weight in the maximal performance of muscle strength (Jimenez-Pavon et al., 2012; Huang et al., 2014a, b).

Clinical examinations included a general physical examination and biochemical blood tests. Height and body weight were measured using a standard protocol, and the body mass index (BMI) was calculated as weight/height^2^ (kg/m^2^). We used a BMI cutoff of 24 based on the Working Group on Obesity in China and the standard of WS/T 428-2013 (China) recommendations for country-specific and ethnicity-specific BMI cutoff in China (Zhou, 2002). Systolic blood pressure (SBP) and diastolic blood pressure (DBP) were measured twice in the right arm using an automatic device (KD598; Andon) after minutes of rest in a seated position. The mean of these two measurements was taken as the final blood pressure (BP). Hypertension was defined as having a BP higher than 140/90 mm Hg (SBP/DBP) or a history of hypertension. Blood samples for the assessment of fasting blood glucose (FBG) and blood lipids were drawn from the antecubital vein, with the participants in a seated position. Specimens were collected in siliconized vacuum plastic tubes. The FBG levels were measured using the glucose oxidase method. Diabetes was defined as having an FBG of ≥7.0 mmol/L, oral glucose tolerance test value of ≥11.1 mmol/L, HbA1c of ≥48 mmol/mol (6.5%), or a history of diabetes, which is in accordance with the latest recommendations from the American Diabetes Association (American Diabetes Association, 2014). As for plasma lipids, plasma triglycerides (TG) and total cholesterol (TC) were measured using enzymatic methods. Plasma low-density lipoprotein cholesterol (LDL-C) was quantified using the polyvinyl sulfuric acid precipitation method, and serum high-density lipoprotein cholesterol (HDL-C) was measured using the chemical precipitation method and appropriate kits (Roche Cobas 8000 modular analyzer, Mannheim, Germany). Hyperlipidemia was defined as TC of ≥5.17 mmol/L, TG of ≥1.7 mmol/L, LDL-C of ≥3.37 mmol/L, or a history of hyperlipidemia.

### Statistical Analysis

Age and sex were specified as sociodemographic risk factors of interest *a priori*. We applied the age- and sex-specific rates of MCI of the China and Tianjin standard populations, obtained from the China Health Statistical Yearbook 2018, to calculate sex- and age-standardized prevalence estimates so that direct comparisons could be made between populations. Associations between the sociodemographic variables, health-related variables, and MCI were examined overall and by age and sex. For the descriptive analysis, an analysis of variance for continuous variables (except for weight-adjusted GS by an analysis of covariance) and a logistic regression analysis for categorical variables were used to compare differences between those with and without MCI. Continuous variables are presented as the geometric mean and 95% confidence interval (CI) after logarithmic transformation. Categorical variables are shown as a number (percentage). For the main analysis, the status of MCI was considered as a dependent variable, and the sociodemographic and health-related variables as independent variables. Continuous variables (e.g., sleepiness and GS) were log-transformed before analysis, except for BMI. Associations between the sociodemographic and health-related variables and the status of MCI were assessed using a multivariate logistic regression in two different models, where the odds ratios (ORs) and 95% CIs were calculated. Specifically, model 1 was adjusted for age and sex, while model 2 was additionally adjusted for all other variables, including education level, income, marital status, employment status, PA, smoking, alcohol drinking, sleep duration, sleepiness, BMI, GS, hypertension, diabetes, and hyperlipidemia. The *P-*values for linear trends were calculated by treating the categorical variables as ordinal variables in the model. We used χ^2^ likelihood-ratio tests to assess the heterogeneity within age and sex categories. General linear models were used to calculate β coefficients and 95% CIs for risk factors related to the MMSE scores in two different models: (1) adjusting for age and sex, (2) additionally adjusting for all other variables.

**FIGURE 2:**
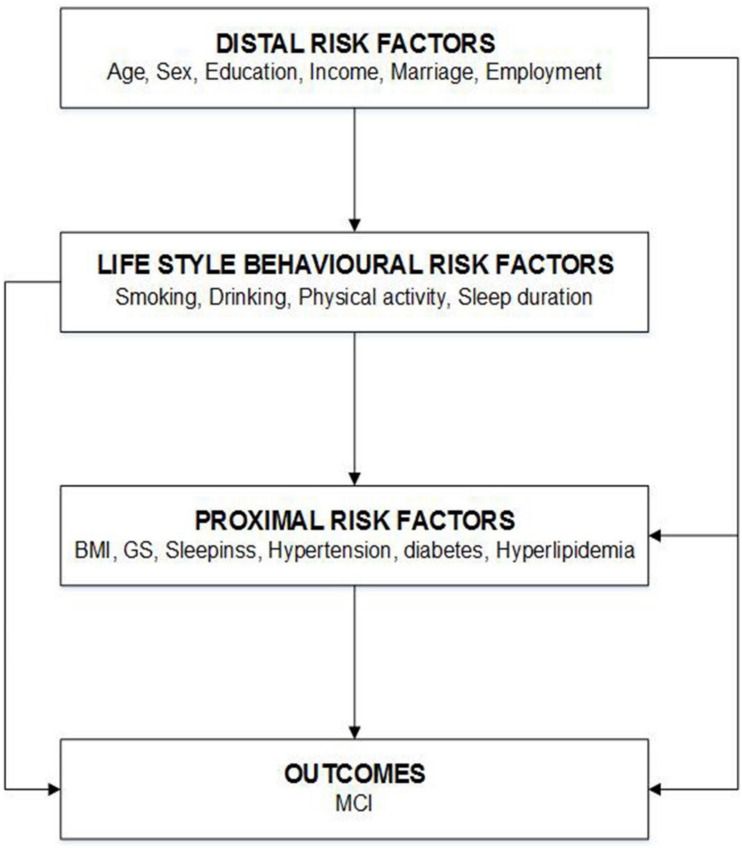
Conceptual model risk factors for mild cognitive impairment (MCI).

For all predefined variables, missing values represented less than 3% of the data of each variable. Information pertinent to the physical examination, including BMI, BP, blood glucose, and blood lipids, was missing for 135 participants. Compared with the participants included in the analytical sample (*n* = 4,631), which is the population who satisfied the inclusion and/or exclusion criteria and were included in the statistical analysis, those excluded due to missing data (*n* = 135) did not differ in terms of sex but were younger and more likely to have lower education levels (Supplementary Table 1). A complete case analysis was conducted as the main analysis, and multiple imputations were performed for missing data in a sensitivity analysis. Multivariate normal imputation was used to impute missing physical examination values (Lee and Carlin, 2010). Moreover, variance inflation factors (VIFs) were used to detect multicolinearity among covariates in the final model. VIFs exceeding 10 were a sign of multicolinearity. To assess the potential for reverse causation, we conducted sensitivity analyses by (1) excluding 362 participants who had changed their lifestyles including diet, drinking, smoking, PA, and sleeping habits, in the past 5 years; (2) excluding 766 participants with long-term medication use; and (3) using a conceptual framework (Price et al., 2018) to categorize the risk factors for MCI into distal factors (e.g., age, sex, education level, income, marital status, and employment), lifestyle behavioral risk factors, and proximal factors (e.g., BMI, GS, and hypertension), assuming the former influenced the latter. This framework determined the factors to retain in the multivariate models. We treated the distal factors as potential confounders of the association between lifestyle behavioral risk factors and the prevalence of MCI. Similarly, the distal and lifestyle behavioral risk factors were considered as potential confounders for the association between proximal risk factors and MCI (Figure 2). SAS version 9.4 (SAS Institute, Inc., Cary, NC, United States) was used for all statistical analyses. A two-sided *P-*value of <0.05 was considered statistically significant.

## Results

### Study Participant Characteristics

**FIGURE 3:**
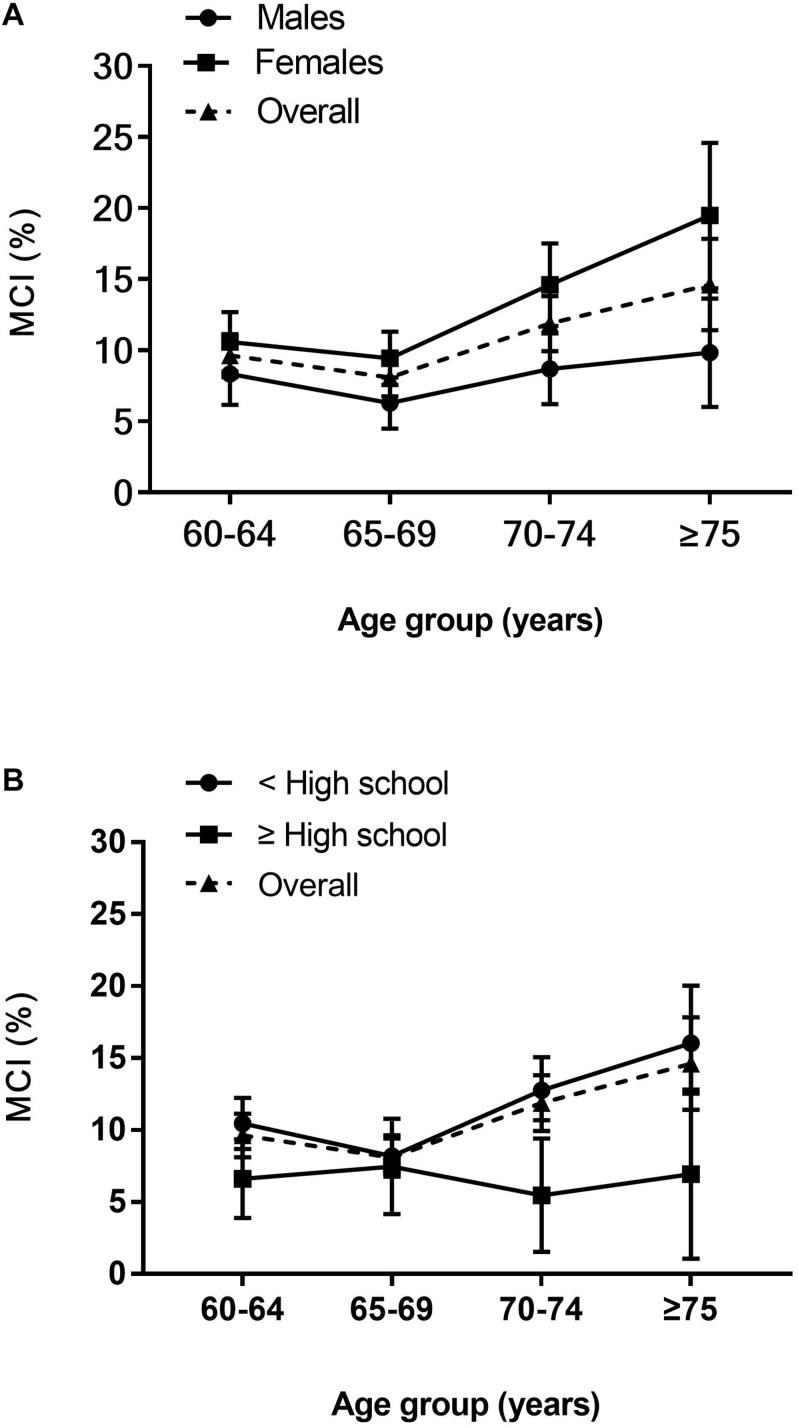
Age-specific prevalence of MCI. **(A)** Age and sex-specific prevalence of MCI. **(B)** Age and education-specific prevalence of MCI. Prevalence point estimates are presented with (vertical) 95% CIs.

Between March 2018 and June 2019, a total of 4,631 individuals were recruited (2,052 males and 2,579 females), of whom 468 (10.1%) were diagnosed with MCI (162 males and 306 females, Table 1). Participants were divided into four age groups, 60–64, 65–69, 70–74, and ≥75 years, with 141 (9.6%), 132 (8.1%), 127 (11.9%), and 68 (14.6%) people, respectively. When standardized to China’s national population, the age- and sex-standardized prevalence of MCI among people aged ≥ 60 years was 10.7% in the study sample. The age-standardized MCI prevalence was slightly higher in females (13.1%) than in males (8.2%). Overall, the sex-standardized MCI prevalence increased with age, with prevalence rates in each age category at 9.5, 7.9, 11.7, and 15.1%, respectively. The prevalence increased more steeply with age in females than males (Figure 3A). Participants with below high school level education had a consistently higher prevalence of MCI than those with an education level of high school or above in all age groups. Meanwhile, the MCI prevalence increased with increasing age in participants with an education level below high school (Figure 3B).

**TABLE 1:** Prevalence of (MCI) in adults aged 60 years and older by age group and sex.

Group	*n*	MCI	Crude prevalence (%)	Standardized prevalence (%)^*a*^	
				Tianjin	China
Participants	4,631	468	10.1	10.7	10.7
**Sex**					
Males	2,052	162	7.9	8.2	8.2
Females	2,579	306	11.9	13.0	13.1
**Age (years)**					
60–64	1,464	141	9.6	9.5	9.5
65–69	1,633	132	8.1	7.9	7.9
70–74	1,069	127	11.9	11.8	11.7
75-∼	465	68	14.6	15.1	15.1

*^*a*^Age- and sex-standardized prevalence.*

Participants’ characteristics are summarized in Table 2. The majority of patients with MCI were female. MCI patients tended to be older and have lower levels of education and income, while non-MCI participants were more likely to be married and working full- or part-time (*P* < 0.05). Regarding health-related variables, a greater proportion of MCI patients had hypertension, longer sleep duration, higher LDL-C, SBP, sleepiness, lower BMI, and GS, and were less likely to be physically active, drinkers, and ex-smokers (*P* < 0.05). Not surprisingly, participants who exhibited MCI displayed a worse cognitive performance on the MMSE (*P* < 0.0001). The descriptive profile of the participants showed some sex and age differences (Tables 3, 4). Males with MCI were more likely to have hypertension and less likely to be married than females (*P* < 0.05). Compared to males, females with MCI had lower education levels, income, BMI, a higher proportion of longer sleep duration, were less likely to have hyperlipidemia and a full- or part-time job, but were more likely to be older and have higher levels of sleepiness (*P* < 0.05). Participants with MCI tended to have lower GS and MMSE scores in all four age groups (*P* < 0.05).

**TABLE 2:** Characteristics of the participants with and without MCI (*n* = 4,631).

Characteristics	MCI		*P-*value^*a*^
	No	Yes	
**No. of subjects**	4,163	468	
**Sociodemographic characteristics**			
**Age group (%, years)**^*c*^			
60–64	1,323 (31.8)	141 (30.1)	0.47
65–69	1,501 (36.1)	132 (28.2)	**<0.001**^**d**^
70–74	942 (22.6)	127 (27.1)	**0.029**
≥75	387 (9.54)	68 (14.5)	**<0.001**
**Sex (males, %)**	1,890 (45.4)	162 (34.6)	**<0.0001**
**Education level (≥high school, %)**	707 (17.0)	51 (10.9)	**<0.001**
**Currently married (%)**	3,673 (88.2)	394 (84.2)	**0.012**
**Income status (%, RMB)**			
<3,000	2,992 (71.9)	363 (77.6)	**<0.01**
3,000–5,000	592 (14.2)	61 (13.0)	0.48
>5,000	579 (13.9)	44 (9.40)	**<0.01**
**Employed (%)**	287 (6.89)	19 (4.06)	**0.021**
**Health-related variables**			
**BMI (kg/m^2^)**	25.6 (25.5, 25.7)^*b*^	25.0 (24.7, 25.3)	**<0.001**
**Hypertension (%)**	2,166 (52.0)	282 (60.3)	**<0.001**
**SBP (mmHg)**	132.4 (132.0, 132.8)	134.6 (133.4, 135.8)	**<0.001**
**DBP (mmHg)**	80.5 (80.3, 80.8)	80.5 (79.9, 81.2)	1.00
**Diabetes (%)**	761 (18.3)	89 (19.0)	0.70
**FBG (mmol/L)**	5.33 (5.30, 5.37)	5.22 (5.11, 5.32)	**0.028**
**Hyperlipidemia (%)**	2,654 (63.8)	278 (59.4)	0.064
**TC (mmol/L)**	5.09 (5.06, 5.12)	5.12 (5.02, 5.22)	0.59
**TG (mmol/L)**	1.41 (1.39, 1.43)	1.35 (1.29, 1.41)	0.070
**LDL-C (mmol/L)**	2.48 (2.45, 2.50)	2.55 (2.49, 2.62)	**0.019**
**HDL-C (mmol/L)**	1.28 (1.27, 1.29)	1.29 (1.27, 1.32)	0.34
**GS (adjusted weight) (kg)**	23.1 (22.8, 23.3)	20.4 (19.7, 21.2)	**<0.0001**
**PA (≥23.0 METs-h/w, %)**	2,700 (64.9)	265 (56.6)	**<0.001**
**Sleepiness (scores)**	9.33 (9.24, 9.43)	10.0 (9.73, 10.3)	**<0.0001**
**Sleep duration (%)**			
<6.5 h	263 (6.32)	23 (4.91)	0.23
6.5–8.5 h	2,266 (54.4)	202 (43.2)	**<0.0001**
>8.5 h	1,634 (39.3)	243 (51.9)	**<0.0001**
**Smoking status (%)**			
Non-smoker	2,648 (63.6)	316 (67.5)	0.095
Ex-smoker	335 (8.05)	21 (4.49)	**<0.01**
Current smoker	1,180 (28.3)	131 (28.0)	0.87
**Alcohol drinking (%)**	3,191 (23.4)	390 (16.7)	**<0.01**
**MMSE scores**	25.9 (25.8, 26.1)	17.8 (17.5, 18.1)	**<0.0001**
**ADL scores**	15.4 (15.3, 15.6)	15.6 (15.3, 15.9)	0.34

*BMI, body mass index; TC, total cholesterol; TG, triglycerides; LDL-C, low-density lipoprotein cholesterol; HDL-C, high-density lipoprotein cholesterol; SBP, systolic blood pressure; DBP, diastolic blood pressure; GS, grip strength; PA, physical activity; METs, metabolic equivalents; MMSE, Mini-Mental State Examination; ADL, activities of daily living. ^*a*^Analysis of variance or logistic regression analysis. ^*b*^Geometric least square mean (95% confidence interval) (all such values). ^*c*^Categorical variables are expressed as number (percentage) of participants. ^*d*^ Boldface indicates statistical significance (*P* < 0.05) (or appropriate value).*

**TABLE 3:** Characteristics of the participants with and without MCI stratified by sex (*n* = 4,631).

Characteristics	Males (*n* = 2,052)		*P-*value^*a*^	Females (*n* = 2,579)		*P*-value
	No	Yes		No	Yes	
**No. of subjects**	1,890	162		2,273	306	
**Sociodemographic characteristics**						
**Age group (%, years)**^*c*^						
60–64	572 (30.3)	52 (32.1)	0.63	751 (33.0)	89 (29.1)	0.17
65–69	656 (34.7)	44 (27.2)	0.053	845 (37.2)	88 (28.8)	**<0.01**
70–74	451 (23.9)	43 (26.5)	0.44	491 (21.6)	84 (27.5)	**0.021**
≥ 75	211 (11.2)	23 (14.2)	0.24	186 (8.18)	45 (14.7)	**<0.001**
**Education level (≥ high school,%)**	478 (25.3)	35 (21.6)	0.30	229 (10.1)	16 (5.23)	**<0.01**
**Currently married (%)**	1,704 (90.2)	136 (84.0)	**0.014**^**d**^	1,969 (86.6)	258 (84.3)	0.27
**Income status (%, RMB)**						
<3,000	1,259 (66.6)	113 (69.8)	0.42	1,733 (76.2)	250 (81.7)	**0.034**
3,000–5,000	295 (15.6)	29 (17.9)	0.44	297 (13.1)	32 (10.5)	0.20
>5,000	336 (17.8)	20 (12.4)	0.082	243 (10.7)	24 (7.8)	0.13
**Employed (%)**	206 (10.9)	15 (9.26)	0.52	81 (3.56)	4 (1.31)	**0.047**
**Health-related variables**						
**BMI (kg/m^2^)**	25.4 (25.3, 25.6)^*b*^	25.1 (24.7, 25.6)	0.19	25.7 (25.5, 25.8)	24.9 (24.6, 25.3)	**<0.001**
**Hypertension (%)**	954 (50.5)	102 (63.0)	**<0.01**	1,212 (53.3)	180 (58.8)	0.070
**SBP (mmHg)**	133.1 (132.5, 133.7)	134.6 (132.7, 136.6)	0.14	131.9 (131.3, 132.4)	134.6 (133.1, 136.1)	**<0.001**
**DBP (mmHg)**	81.4 (81.0, 81.7)	81.9 (80.7, 83.0)	0.43	79.9 (79.6, 80.2)	79.9 (79.0, 80.7)	0.98
**Diabetes (%)**	319 (16.9)	30 (18.5)	0.59	442 (19.5)	59 (19.3)	0.95
**FBG (mmol/L)**	5.33 (5.28, 5.38)	5.21 (5.04, 5.37)	0.18	5.34 (5.30, 5.39)	5.22 (5.10, 5.35)	0.078
**Hyperlipidemia (%)**	994 (52.6)	85 (52.5)	0.98	1,660 (70.0)	193 (63.1)	**<0.001**
**TC (mmol/L)**	4.85 (4.80, 4.90)	4.87 (4.71, 5.04)	0.77	5.30 (5.26, 5.35)	5.26 (5.13, 5.38)	0.49
**TG (mmol/L)**	1.24 (1.21, 1.27)	1.26 (1.17, 1.36)	0.66	1.57 (1.54, 1.60)	1.40 (1.32, 1.47)	**<0.0001**
**LDL-C (mmol/L)**	2.36 (2.33, 2.39)	2.43 (2.33, 2.54)	0.15	2.58 (2.55, 2.61)	2.62 (2.54, 2.70)	0.32
**HDL-C (mmol/L)**	1.26 (1.24, 1.28)	1.24 (1.19, 1.30)	0.52	1.30 (1.29, 1.31)	1.32 (1.29, 1.36)	0.12
**GS (adjusted weight) (kg)**	31.3 (30.8, 31.7)	28.9 (27.6, 30.3)	**<0.0001**	18.1 (17.8, 18.3)	16.0 (15.4, 16.6)	**<0.0001**
**PA(≥ 23.0 METs-h/w,%)**	1,235 (65.3)	91 (56.2)	**0.020**	1,465 (64.5)	174 (56.9)	**<0.01**
**Sleepiness (scores)**	9.32 (9.18, 9.45)	10.4 (9.85, 10.9)	**<0.0001**	9.35 (9.22, 9.48)	9.85 (9.49, 10.22)	**<0.01**
**Sleep duration (%)**						
<6.5 h	121 (6.40)	9 (5.56)	0.67	142 (6.25)	14 (4.58)	0.25
6.5–8.5 h	1,072 (56.7)	79 (48.8)	0.051	1,194 (52.5)	123 (40.2)	**<0.0001**
>8.5 h	697 (36.9)	74 (45.7)	**0.027**	937 (41.2)	169 (55.2)	**<0.0001**
**Smoking status (%)**						
Non-smoker	707 (37.4)	57 (35.2)	0.57	1,941 (85.4)	259 (84.6)	0.73
Ex-smoker	286 (15.1)	16 (9.88)	0.075	50 (2.20)	5 (1.63)	0.52
Current smoker	898 (47.5)	89 (54.9)	0.071	282 (12.4)	42 (13.7)	0.51
**Alcohol drinking (%)**	981 (48.1)	92 (43.2)	0.23	2,210 (2.77)	298 (2.61)	0.87
**MMSE scores**	27.2 (27.1, 27.3)	20.7 (20.4, 21.1)	**<0.0001**	24.8 (24.6, 25.0)	16.4 (16.1, 16.8)	**<0.0001**
**ADL scores**	15.2 (15.0, 15.3)	15.0 (14.5, 15.5)	0.40	15.7 (15.5, 15.8)	16.0 (15.5, 16.4)	0.19

*BMI, body mass index; TC, total cholesterol; TG, triglycerides; LDL-C, low-density lipoprotein cholesterol; HDL-C, high-density lipoprotein cholesterol; SBP, systolic blood pressure; DBP, diastolic blood pressure; FBG, fasting blood glucose; GS, grip strength; PA, physical activity; METs, metabolic equivalents; MMSE, Mini-Mental State Examination; ADL, activities of daily living. ^*a*^Analysis of variance or logistic regression analysis. ^*b*^Geometric least square mean (95% confidence interval) (all such values). ^*c*^Categorical variables are expressed as number (percentage) of participants. ^*d*^Boldface indicates statistical significance (*P* < 0.05) (or appropriate value).*

**TABLE 4:** Characteristics of the participants with and without MCI among the different age groups (*n* = 4,631)^*a*^.

Characteristics	60–64 (*n* = 1,464)		65–69 (*n* = 1,633)		70–74 (*n* = 1,069)		≥75 (*n* = 465)	
	No	Yes	No	Yes	No	Yes	No	Yes
**No. of subjects**	1,323	141	1,501	132	942	127	397	68
**Sociodemographic characteristics**								
**Sex (males,%)**^*d,e,f,g*^	751 (43.2)	89 (36.9)	845 (43.7)	88 (33.3)	491 (47.9)	84 (33.9)	186 (53.2)	45 (33.8)
**Education level (≥ high school,%)**^*c*,*e*^	296 (22.4)	21 (14.9)	223 (14.9)	18 (13.6)	121 (12.9)	7 (5.51)	67 (16.9)	5 (7.35)
**Currently married (%)**^*e*^	1,210 (91.5)	126 (89.4)	1,352 (90.1)	122 (92.4)	814 (86.4)	100 (78.7)	297 (74.8)	46 (67.7)
**Income status (%, RMB)**								
<3,000^*e*^	917 (69.3)	106 (75.2)	1,091 (72.7)	91 (68.9)	677 (71.9)	109 (85.8)	307 (77.3)	57 (83.8)
3,000–5,000	197 (14.9)	23 (16.3)	210 (14.0)	21 (15.9)	140 (14.86)	12 (9.45)	45 (11.34)	5 (7.35)
>5,000^*c,e*^	209 (15.8)	12 (8.51)	200 (13.3)	20 (15.2)	125 (13.3)	6 (4.72)	45 (11.3)	6 (8.82)
**Employed (%)**^*c*^	152 (11.5)	7 (4.96)	100 (6.66)	11 (8.33)	28 (2.97)	1 (0.79)	7 (1.76)	0 (0)
**Health-related variables**								
**BMI (kg/m^2^)**^*e,f*^	25.6 (25.4, 25.8)^*b*^	25.4 (24.9, 26.0)	25.6 (25.5, 25.8)	25.7 (25.2, 26.3)	25.6 (25.3, 25.8)	24.6 (24.0, 25.1)	25.2 (24.9, 25.6)	23.6 (22.9, 24.3)
**Hypertension (%)**^*e*^	601 (45.4)	74 (52.5)	814 (54.2)	77 (54.2)	536 (56.9)	88 (69.3)	215 (54.2)	43 (63.2)
**SBP (mmHg)**^*e*^	130.2 (129.5, 130.8)	131.6 (129.5, 133.7)	132.6 (132.0, 133.3)	133.8 (131.5, 136.0)	134.4 (133.6, 135.2)	138.0 (135.7, 140.4)	134.6 (133.4, 135.8)	136.2 (133.3, 139.3)
**DBP (mmHg)**	80.5 (80.1, 80.9)	80.3 (79.1, 81.5)	80.7 (80.4, 81.1)	80.9 (79.7, 82.2)	80.7 (80.2, 81.2)	80.6 (79.3, 82.0)	79.8 (79.1, 80.4)	80.1 (78.5, 81.8)
**Diabetes (%)**	224 (16.9)	29 (20.6)	275 (18.3)	29 (22.0)	188 (20.0)	25 (19.7)	74 (18.6)	6 (8.82)
**FBG (mmol/L)**^*e,f*^	5.29 (5.24, 5.35)	5.28 (5.11, 5.47)	5.34 (5.29, 5.40)	5.40 (5.21, 5.60)	5.38 (5.30, 5.45)	5.10 (4.91, 5.30)	5.34 (5.24, 5.45)	4.95 (4.72, 5.19)
**Hyperlipidemia (%)**^*c*^	882 (66.7)	82 (58.2)	949 (63.2)	85 (64.4)	576 (61.2)	75 (59.1)	247 (62.2)	36 (52.9)
**TC (mmol/L)**	5.13 (5.07, 5.19)	5.11 (4.93, 5.30)	5.06 (5.00, 5.11)	5.27 (5.07, 5.47)	5.10 (5.03, 5.17)	5.04 (4.85, 5.23)	5.08 (4.98, 5.19)	5.01 (4.77, 5.28)
**TG (mmol/L)**	1.44 (1.40, 1.48)	1.35 (1.24, 1.46)	1.44 (1.40, 1.47)	1.45 (1.33, 1.58)	1.36 (1.32, 1.40)	1.30 (1.19, 1.42)	1.33 (1.27, 1.39)	1.26 (1.13, 1.41)
**LDL-C (mmol/L)**	2.50 (2.46, 2.54)	2.57 (2.46, 2.69)	2.45 (2.41, 2.48)	2.56 (2.44, 2.69)	2.48 (2.44, 2.53)	2.54 (2.43, 2.66)	2.48 (2.42, 2.55)	2.53 (2.37, 2.71)
**HDL-C (mmol/L)**	1.29 (1.28, 1.31)	1.29 (1.24, 1.33)	1.26 (1.24, 1.27)	1.29 (1.24, 1.35)	1.30 (1.27, 1.32)	1.31 (1.24, 1.38)	1.28 (1.25, 1.31)	1.29 (1.22, 1.37)
**GS (adjusted weight) (kg)**^*c,d,e,f*^	24.9 (24.4, 25.5)	22.5 (21.1, 24.0)	23.1 (22.7, 23.5)	20.5 (19.3, 21.9)	21.5 (21.0, 22.1)	19.3 (18.0, 20.7)	21.0 (20.2, 21.9)	18.0 (16.2, 20.0)
**PA (≥23.0 METs-h/w, %)**^*c,e*^	881 (66.6)	82 (58.2)	984 (65.6)	80 (60.6)	600 (63.7)	64 (50.4)	235 (59.2)	39 (57.4)
**Sleepiness (scores)**^*c,d*^	9.35 (9.18, 9.51)	10.5 (9.96, 11.1)	9.29 (9.14, 9.45)	9.97 (9.43, 10.5)	9.45 (9.25, 9.65)	9.77 (9.22, 10.4)	9.18 (8.89, 9.49)	9.60 (8.87, 10.4)
**Sleep duration (%)**								
<6.5 h	85 (6.42)	8 (5.67)	103 (6.86)	9 (6.82)	55 (5.84)	3 (2.36)	20 (5.04)	3 (4.41)
6.5–8.5 h^*d,e*^	779 (58.9)	81 (57.5)	858 (57.2)	59 (44.7)	459 (48.7)	38 (29.9)	170 (42.8)	24 (35.3)
>8.5 h^*d,e*^	459 (34.7)	52 (36.9)	540 (36.0)	64 (48.5)	428 (45.4)	86 (67.7)	207 (52.1)	41 (60.3)
**Smoking status (%)**								
Non-smoker	839 (63.4)	92 (65.3)	955 (63.6)	87 (65.9)	601 (63.8)	89 (70.1)	253 (63.7)	48 (70.6)
Ex-smoker^*f*^	88 (6.65)	7 (4.96)	120 (7.99)	7 (5.30)	87 (9.24)	6 (4.72)	40 (10.1)	1 (1.47)
Current smoker	396 (29.9)	42 (29.8)	426 (28.4)	38 (28.8)	254 (27.0)	32 (25.2)	104 (26.2)	19 (27.9)
**Drinking status (Yes, %)**^*d*^	983 (25.7)	112 (20.6)	1,166 (22.3)	113 (14.4)	733 (22.2)	108 (15.0)	309 (22.2)	57 (16.2)
**MMSE scores**^*c,d,e,f*^	26.2 (26.0, 26.4)	18.4 (17.9, 18.8)	25.7 (25.5, 25.9)	18.2 (17.6, 18.7)	26.0 (25.7, 26.3)	17.5 (17.0, 18.1)	25.2 (24.74, 25.7)	16.6 (15.8, 17.4)
**ADL scores**	15.1 (14.9, 15.3)	15.1 (14.7, 15.6)	15.4 (15.2, 15.6)	15.7 (15.1, 16.4)	15.6 (15.4, 15.8)	15.6 (15.0, 16.3)	16.5 (16.0, 17.0)	16.3 (15.2, 17.4)

*BMI, body mass index; TC, total cholesterol; TG, triglycerides; LDL-C, low-density lipoprotein cholesterol; HDL-C, high-density lipoprotein cholesterol; SBP, systolic blood pressure; DBP, diastolic blood pressure; FBG, fasting blood glucose; GS, grip strength; PA, physical activity; METs, metabolic equivalents; MMSE, Mini-Mental State Examination; ADL, Activities of Daily Living. ^*a*^Analysis of variance or logistic regression analysis. ^*b*^Geometric least square mean (95% confidence interval) (all such values). ^*c*^*P* < 0.05 for group of 60 to 64 years; ^*d*^*P* < 0.05 for group of 65 to 69 years; ^*e*^*P* < 0.05 for group of 70–74 years; ^*f*^*P* < 0.05 for group of aged 75 and over. ^*g*^Categorical variables are expressed as number (percentage) of participants.*

### Association Between the Sociodemographic Variables and MCI

**FIGURE 4:**
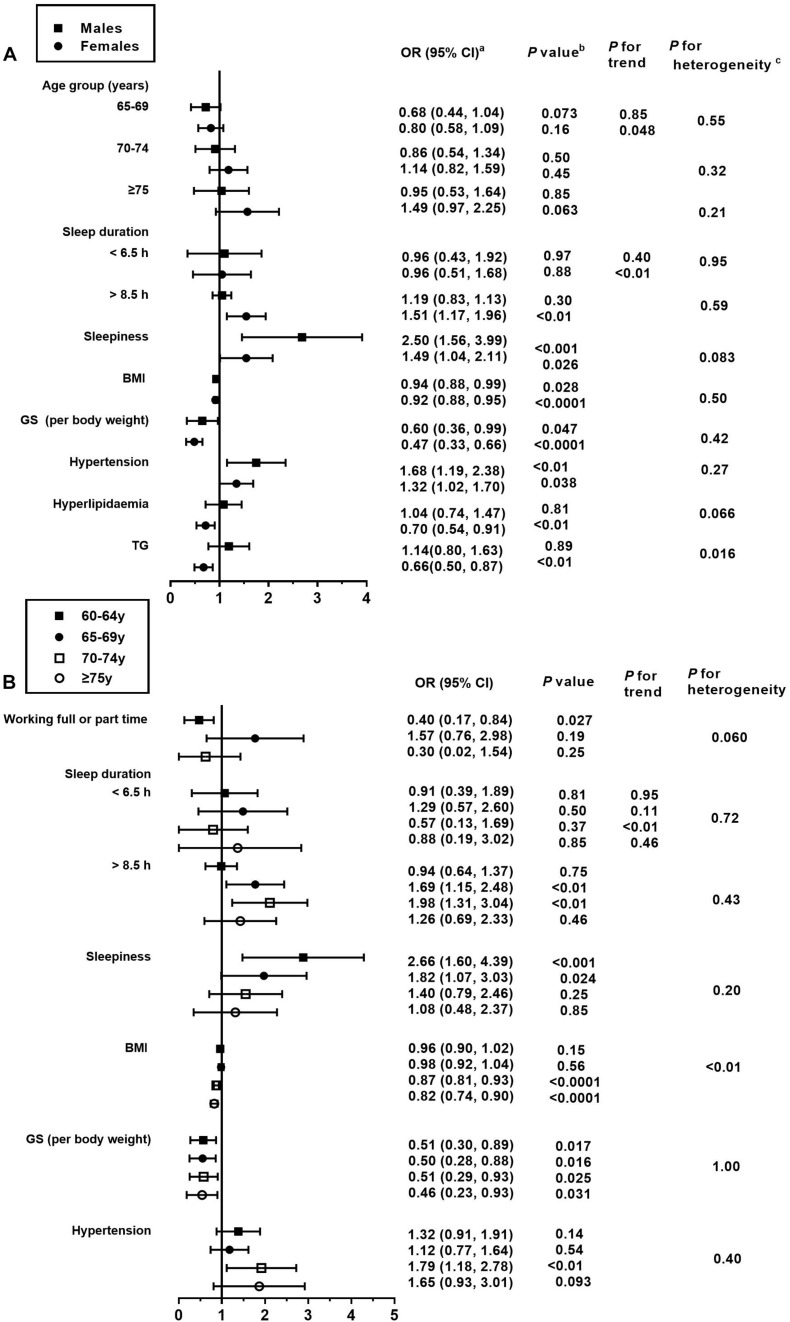
Association between the risk factors and MCI, stratified by sex and age. BMI, body mass index; GS, grip strength; TG, triglyceride. This figure only shows the risk factors that are significantly associated with MCI. For details on the association of all risk factors with MCI, please refer to Supplementary Tables 3, 4. (a) Fully adjusted models are adjusted for age, sex, education level, income status, marital status, employment status, physical activity, smoking status, drinking status, sleep duration, BMI, GS, hypertension, diabetes, hyperlipidemia or its diagnostic indicators, and sleepiness. (b) Obtained using a multiple logistic regression analysis. (c) Comparison between the odds ratios associated with sex **(A)** and age groups **(B)** using *P* for heterogeneity.

After multivariate adjustment, participants in the second age group (65–69 years) were significantly associated with MCI when compared with individuals aged 60–64 years (OR = 0.74; 95% CI: 0.58, 0.96; Table 5). No significant association was seen between other sociodemographic variables and MCI. Intriguingly, in the youngest age group, participants who worked full- or part-time appeared to have a lower prevalence of MCI relative to those who were retired or not working (OR = 0.40; 95% CI: 0.17, 0.84; Figure 4B).

**TABLE 5:** Association between the risk factors and MCI (adjusted ORs and 95% confidence intervals; *n* = 4,631).

Variables	Age and sex adjusted		Multivariable adjusted^*b*^	
	OR (95%CI)	*P*-value^*a*^	OR (95%CI)	*P*-value
**Sociodemographic characteristics**				
**Age group (years)**				
60–64	1.00 (reference)	–	1.00 (reference)	–
65–69	0.83 (0.64, 1.06)	0.13	**0.74 (0.58, 0.96)**	**0.021**
70–74	1.29 (1.00, 1.66)	0.052	1.00 (0.76, 1.30)	0.98
≥75	**1.67 (1.22, 2.27)**	**<0.01**^**c**^	1.20 (0.86, 1.67)	0.28
***P* for trend**^*a*^	**<0.001**		0.25	
**Females vs. males**	**1.61 (1.32, 1.97)**	**<0.0001**	1.11 (0.83, 1.49)	0.47
**High school and above**	**0.68 (0.49, 0.92)**	**0.014**	0.81 (0.58, 1.13)	0.23
**Income status (RMB)**				
<3,000	1.00 (reference)	–	1.00 (reference)	–
3,000–5, 000	0.90 (0.67, 1.19)	0.46	1.14 (0.84, 1.53)	0.39
>5,000	**0.69 (0.49, 0.94)**	**0.024**	0.94 (0.65, 1.33)	0.71
***P* for trend**	**0.02**		>0.99	
**Married vs. unmarried**	0.81 (0.62, 1.07)	0.12	0.87 (0.67, 1.16)	0.34
**Working vs. no work**	0.72 (0.43, 1.13)	0.17	0.69 (0.41, 1.10)	0.14
**Health-related variables**				
**PA (METs-h/w) (**≥23.0 vs. <23.0)	**0.72 (0.60, 0.88)**	**<0.01**	**0.79 (0.64, 0.96)**	**0.018**
**Smoking status**				
Non-smoker	1.00 (reference)	−	1.00 (reference)	–
Ex-smoker	0.69 (0.42, 1.10)	0.13	0.65 (0.39, 1.03)	0.077
Current smoker	1.20 (0.94, 1.52)	0.15	1.21 (0.94, 1.56)	0.14
***P* for trend**	0.16		0.15	
**Alcohol drinking**	0.86 (0.64, 1.16)	0.32	0.85 (0.62, 1.16)	0.31
**Sleep duration (h)**				
<6.5 h	0.98 (0.61, 1.50)	0.91	0.94 (0.58, 1.46)	0.83
6.5–8.5 h	1.00 (reference)	–	1.00 (reference)	–
>8.5 h	**1.56 (1.28, 1.90)**	**<0.0001**	**1.40 (1.14, 1.72)**	**<0.01**
***P* for trend**	**<0.001**		**0.006**	
**Sleepiness (scores)**	**1.86 (1.41, 2.44)**	**<0.0001**	**1.80 (1.36, 2.37)**	**<0.0001**
**BMI (kg/m^2^)**	**0.95 (0.92, 0.98)**	**<0.001**	**0.92 (0.89, 0.95)**	**<0.0001**
**GS(per body weight) (kg/kg)**	**0.54 (0.41, 0.71)**	**<0.0001**	**0.50 (0.38, 0.67)**	**<0.0001**
**Hypertension**	**1.37 (1.12, 1.67)**	**<0.01**	**1.44 (1.18, 1.77)**	**<0.001**
**Diabetes**	1.03 (0.80, 1.31)	0.79	1.01 (0.78, 1.30)	0.92
**Hyperlipidemia**	**0.75 (0.62, 0.92)**	**<0.01**	0.83 (0.67, 1.02)	0.070

*BMI, body mass index; GS, grip strength; PA, physical activity; METs, metabolic equivalents. ^*a*^Obtained by using multiple logistic regression analysis. ^*b*^Additionally adjusted for education level, income status, marriage status, employment status, physical activity, smoking status, drinking status, sleep duration, BMI, GS, hypertension, diabetes, hyperlipidemia, sleepiness. ^*c*^Boldface indicates statistical significance (*P* < 0.05) (or appropriate value).*

### Association Between Health-Related Variables and Mild Cognitive Impairment

In the present study, BMI was inversely associated with MCI in both the adjusted models, where the ORs (95% CI) were 0.95 (0.92, 0.98) and 0.92 (0.89, 0.95), respectively (Table 5). Similar associations were observed when males and females were analyzed separately (*P* for heterogeneity = 0.50; Figure 4A and Supplementary Table 4). Moreover, the magnitude of the inverse association between BMI and MCI increased with age (*P* for heterogeneity = 0.003; Figure 4B and Supplementary Table 5). Additionally, when we modeled BMI as a categorical variable, the OR (95% CI) of developing MCI was 0.68 (0.55, 0.85) for participants with a BMI of ≥24 kg/m^2^ compared to those with a BMI of <24 kg/m^2^. We found that hyperlipidemia was significantly associated with a lower prevalence of MCI in females only in the stratified analyses by sex and age (OR = 0.70; 95% CI: 0.54, 0.91). Moreover, the magnitude of the association between TG and MCI was different between the sexes (*P* for heterogeneity = 0.016). In this study, GS (OR = 0.50; 95% CI: 0.38, 0.67) and PA (OR = 0.79; 95% CI: 0.64, 0.96) levels were negatively associated with the presence of MCI in all models. In contrast, the presence of hypertension (OR = 1.44; 95% CI: 1.18, 1.77) was positively associated with the prevalence of MCI in all models (Table 5). As for the age- and sex-specific associations, the GS–MCI relationship in all age groups and the associations of MCI with PA and hypertension in the older age group (70–74 years) remained statistically significant after multivariate adjustment (*P* < 0.05; Figure 4B). GS and hypertension were significantly associated with MCI in both sexes (*P* < 0.05; Figure 4A). The prevalence of MCI increased with higher levels of sleepiness (OR = 1.80; 95% CI: 1.36, 2.37) and sleep duration (>8.5 h vs. 6.5 to 8.5 h) (OR = 1.40; 95% CI: 1.14, 1.72) (Table 5). The former association remained significant in both sexes and the younger age group (60–69 years), and the latter association remained significant in females and the older age group (65–74 years) after multivariate adjustment (*P* for heterogeneity > 0.05; Figures 4A,B).

### Association Between Multivariate Factors and the MMSE Score

In the final multivariate models, those with a higher education level, higher income level, married, and currently working had an increase in the MMSE score from 0.55–1.52 as per unit increase of the respective variable (*P* < 0.05).

In contrast, the cognitive scores tended to decrease, ranging from 0.69 to 1.48 among females and those in the older age groups (*P* < 0.05; Table 6). For the abovementioned health-related variables, those with a higher BMI, higher GS, and hyperlipidemia showed an increase of 0.47 to 2.91 on average in the cognitive scores (*P* < 0.05). In contrast, smokers, those with hypertension, higher levels of sleepiness, and higher sleep duration showed a decrease of 0.27–0.85 on average in the cognitive scores (*P* < 0.05). All VIFs ranged from 1.03 to 2.54, indicating no colinearity issues. Similar results were also observed in the sensitivity analyses (Table 7 and Supplementary Table 2). However, the positive association of female sex and older age with MCI appeared enhanced in multivariate models determined by the conceptual framework (Table 8). In the analysis where multiple imputations were used to impute missing values, the results are marginally unchanged (Supplementary Table 3).

**TABLE 6:** Association of the risk factors and MMSE scores in the total population (*n* = 4,631).

Variables	Age and sex adjusted			Multivariable adjusted^*b*^		
	β (95% CI)	*SE*	*P*-value^*a*^	β (95% CI)	*SE*	*P* value
**Sociodemographic characteristics**						
**Age group**						
60–64	(reference group)	**−**	**−**	(reference group)	**−**	**−**
65–69	-0.25 (-0.53, 0.03)	0.14	0.075	0.07 (-0.20, 0.33)	0.14	0.61
70–74	−0.47 (−0.79, −0.16)	0.16	**<0.01**^**c**^	0.22 (−0.08, 0.52)	0.15	0.16
≥ 75	−1.57 (−1.98, −1.15)	0.21	**<0.0001**	−0.69 (−1.09, −0.28)	0.21	**<0.001**
**Sex (females vs. males)**	−2.67 (−2.89, −2.44)	0.12	**<0.0001**	−1.48 (−0.80, −1.16)	0.16	**<0.0001**
**High school and above**	2.33 (2.02, 2.64)	0.16	**<0.0001**	1.52 (1.19, 1.85)	0.17	**<0.0001**
**Income status (RMB)**						
<3,000	(reference group)	−	−	(reference group)	−	−
3,000–5,000	1.38 (1.05, 1.70)	0.17	**<0.0001**	0.74 (0.42, 1.07)	0.16	**<0.0001**
>5,000	2.19 (1.86, 2.53)	0.17	**<0.0001**	1.07 (0.72, 1.42)	0.18	**<0.0001**
**Marital status (Unmarried vs. married)**	0.91 (0.56, 1.26)	0.18	**<0.0001**	0.55 (0.22, 0.89)	0.17	**<0.01**
**Employment status (Working vs. No work)**	0.046 (-0.42, 0.52)	0.24	0.85	0.20 (−0.25, 0.64)	0.23	0.38
**Health related variables**						
**PA (METs-h/w)** (≥23.0 vs. <23.0)	0.70 (0.47, 0.94)	0.12	**<0.0001**	0.31 (0.08, 0.53)	0.12	**<0.01**
**Smoking status**						
Non-smoker	(reference group)	−	−	(reference group)	−	−
Ex-smoker	0.062 (−0.40, 0.52)	0.24	0.79	0.24 (−0.19, 0.68)	0.22	0.28
Current smoker	−0.45 (−0.73, −0.16)	0.15	**<0.001**	−0.35 (−0.63, −0.07)	0.14	**0.013**
**Alcohol drinking**	0.18 (−0.14, 0.51)	0.16	0.26	0.24 (−0.07, 0.55)	0.16	0.13
**Sleep duration (h)**						
<6.5 h	−0.26 (−0.73, 0.22)	0.24	0.30	−0.32 (−0.78, 0.14)	0.23	0.17
6.5–8.5 h	(reference group)	−	−	(reference group)	−	−
>8.5 h	−1.28 (−1.52, −1.05)	0.12	**<0.0001**	−0.85 (−1.08, −0.62)	0.12	**<0.0001**
**Sleepiness (scores)**	−0.95 (−1.30, −0.60)	0.18	**<0.0001**	−0.71 (−1.04, −0.38)	0.17	**<0.0001**
**BMI (kg/m^2^)**	1.61 (0.70, 2.51)	0.46	**<0.001**	2.91 (1.99, 3.84)	0.47	**<0.001**
**GS(per body weight) (kg/kg)**	1.94 (1.60, 2.29)	0.18	**<0.0001**	1.99 (1.64, 2.34)	0.18	**<0.0001**
**Hypertension**	−0.34 (−0.57, −0.11)	0.12	**<0.01**	−0.27 (−0.49, −0.05)	0.11	**0.017**
**Diabetes**	0.05 (−0.24, 0.34)	0.15	0.74	−0.03 (−0.31, 0.26)	0.14	0.86
**Hyperlipidemia**	0.80 (0.56, 1.04)	0.12	**<0.0001**	0.47 (0.24, 0.70)	0.12	**<0.0001**

*BMI, body mass index; GS, grip strength; PA, physical activity; METs, metabolic equivalents; MMSE, Mini-Mental State Examination. ^*a*^Obtained by using generalized linear model. ^*b*^Additionally adjusted for education level, income status, marriage status, employment status, physical activity, smoking status, drinking status, sleep duration, BMI, GS, hypertension, diabetes, hyperlipidemia, sleepiness. ^*c*^Boldface indicates statistical significance (*P* < 0.05) (or appropriate value).*

**TABLE 7:** Association between the risk factors and MCI after excluding participants who had changed their lifestyle in the past 5 years (adjusted ORs and 95% CIs; *n* = 4,242).

Variables	Age and sex adjusted		Multivariable adjusted^*b*^	
	OR (95%CI)	*P*-value^*a*^	OR (95%CI)	*P*-value
**Sociodemographic characteristics**				
**Age group (years)**				
60–64	1.00 (reference)	–	1.00 (reference)	–
65–69	0.81 (0.62, 1.04)	0.10	**0.73 (0.56, 0.94)**	**0.017**
70–74	1.24 (0.95, 1.61)	0.11	0.96 (0.73, 1.27)	0.78
≥75	**1.69 (1.22, 2.31)**^**c**^	**<0.01**	1.20 (0.85, 1.69)	0.29
***P* for trend**^*a*^	**< 0.001**		0.31	
**Females vs. males**	**1.67 (1.36, 2.07)**	**<0.0001**	1.18 (0.87, 1.60)	0.29
**High school and above**	**0.68 (0.49, 0.92)**	**0.016**	0.82 (0.57, 1.15)	0.25
**Income status (RMB)**				
<3,000	1.00 (reference)	–	1.00 (reference)	–
3,000–5,000	0.93 (0.69, 1.24)	0.63	1.19 (0.87, 1.60)	0.26
>5,000	**0.68 (0.48, 0.95)**	**0.029**	0.94 (0.64, 1.35)	0.74
***P* for trend**	**0.035**		0.90	
**Married vs. unmarried**	0.82 (0.62, 1.09)	0.16	0.88 (0.66, 1.18)	0.38
**Working vs. no work**	0.71 (0.42, 1.14)	0.18	0.68 (0.40, 1.10)	0.14
**Health-related variables**				
**PA (METs × h/w)** (≥23.0 vs. <23.0)	**0.76 (0.62, 0.93)**	**<0.01**	**0.81 (0.66, 1.00)**	**0.049**
**Smoking status**				
Non-smoker	1.00 (reference)	–	1.00 (reference)	–
Ex-smoker	0.63 (0.35, 1.06)	0.098	0.61 (0.34, 1.04)	0.087
Smoker	1.18 (0.92, 1.52)	0.19	1.21 (0.93, 1.57)	0.16
***P* for trend**	0.20		0.17	
**Alcohol drinking**	0.92 (0.67, 1.25)	0.58	0.92 (0.66, 1.26)	0.59
**Sleep duration (h)**				
<6.5 h	0.93 (0.57, 1.46)	0.76	0.90 (0.54, 1.42)	0.66
6.58.5 h	1.00 (reference)	–	1.00 (reference)	–
>8.5 h	**1.52 (1.24, 1.87)**	**<0.0001**	**1.37 (1.11, 1.70)**	**<0.01**
***P* for trend**	**<0.001**		**<0.01**	
**Sleepiness (scores)**	**1.79 (1.34, 2.39)**	**<0.0001**	**1.72 (1.28, 2.30)**	**<0.001**
**BMI (kg/m^2^)**	**0.95 (0.92, 0.98)**	**<0.001**	**0.93 (0.89, 0.96)**	**<0.0001**
**GS (per body weight) (kg/kg)**	**0.51 (0.39, 0.68)**	**<0.0001**	**0.47 (0.35, 0.64)**	**<0.0001**
**Hypertension**	**1.35 (1.11, 1.66)**	**<0.01**	**1.44 (1.16, 1.78)**	**<0.001**
**Diabetes**	0.99 (0.76, 1.28)	0.96	0.98 (0.75, 1.27)	0.89
**Hyperlipidemia**	**0.71 (0.58, 0.88)**	**<0.01**	**0.79 (0.64, 0.98)**	**0.031**

*BMI, body mass index; GS, grip strength; PA, physical activity; METs, metabolic equivalents. ^*a*^Obtained by using multiple logistic regression analysis. ^*b*^Additionally adjusted for education level, income status, marriage status, employment status, physical activity, smoking status, drinking status, sleep duration, BMI, GS, hypertension, diabetes, hyperlipidemia, sleepiness. ^*c*^Boldface indicates statistical significance (*P* < 0.05) (or appropriate value).*

**TABLE 8:** Association between the risk factors and MCI: age- and sex-adjusted as well as multivariate-adjusted risk factors (adjusted ORs and 95% CIs; n = 4,631).

Variables	Age and sex adjusted		Multiple factor adjusted	
	OR (95%CI)	*P*-value^*a*^	OR (95%CI)	*P*-value
**Distal risk factors**^*b*^				
**Age group (years)**				
**60–64**	1.00 (reference)	–	1.00 (reference)	–
**65–69**	0.83 (0.64, 1.06)	0.13	0.80 (0.62, 1.02)	0.075
**70–74**	1.29 (1.00, 1.66)	0.052	1.20 (0.93, 1.56)	0.17
**≥75**	**1.67 (1.22, 2.27)**^**e**^	**<0.01**	**1.52 (1.10, 2.08)**	**0.011**
***P* for trend**^*a*^	**<0.001**		**<0.01**	
**Females vs. males**	**1.61 (1.32, 1.97)**	**<0.0001**	**1.47 (1.20, 1.81)**	**<0.001**
**High school and above**	**0.68 (0.49, 0.92)**	**0.014**	0.74 (0.52, 1.02)	0.073
***P* for trend**			0.21	
**Income status (RMB)**				
**<3,000**	1.00 (reference)	–	1.00 (reference)	–
**3,000–5,000**	0.90 (0.67, 1.19)	0.46	0.95 (0.71, 1.27)	0.74
**>5,000**	**0.69 (0.49, 0.94)**	**0.024**	0.78 (0.54, 1.09)	0.16
***P* for trend**	**0.023**		0.18	
**Married vs. unmarried**	0.81 (0.62, 1.07)	0.12	0.83 (0.64, 1.10)	0.20
**Working vs. No work**	0.72 (0.43, 1.13)	0.17	0.69 (0.41, 1.08)	0.13
**Life style behavior risk factors**^*c*^				
**PA (METs × h/w)** (≥ 23.0 vs. <23.0)	**0.72 (0.60, 0.88)**	**<0.01**	**0.78 (0.64, 0.96)**	**0.015**
**Smoking status**				
**Non-smoker**	1.00 (reference)	–	1.00 (reference)	–
**Ex-smoker**	0.69 (0.42, 1.10)	0.13	0.66 (0.40, 1.05)	0.092
**Current smoker**	1.20 (0.94, 1.52)	0.15	1.20 (0.93, 1.53)	0.15
***P* for trend**	0.16		0.29	
**Alcohol drinking**	0.86 (0.64, 1.16)	0.32	0.84 (0.61, 1.13)	0.25
**Sleep duration (h)**				
<6.5 h	0.98 (0.61, 1.50)	0.91	0.95 (0.59, 1.47)	0.83
6.5–8.5 h	1.00 (reference)	–	1.00 (reference)	–
>8.5 h	**1.56 (1.28, 1.90)**	**<0.0001**	**1.48 (1.21, 1.81)**	**<0.01**
***P* for trend**	**<0.001**		**<0.01**	
**Proximal risk factors**^*d*^				
**Sleepiness (scores)**	**1.86 (1.41, 2.44)**	**<0.0001**	**1.80 (1.36, 2.37)**	**<0.0001**
**BMI (kg/m^2^)**	**0.95 (0.92, 0.98)**	**<0.001**	**0.92 (0.89, 0.95)**	**<0.0001**
**GS(per body weight) (kg/kg)**	**0.54 (0.41, 0.71)**	**<0.0001**	**0.50 (0.38, 0.67)**	**<0.0001**
**Hypertension**	**1.37 (1.12, 1.67)**	**<0.01**	**1.44 (1.18, 1.77)**	**<0.001**
**Diabetes**	1.03 (0.80, 1.31)	0.79	1.01 (0.78, 1.30)	0.92
**Hyperlipidemia**	**0.75 (0.62, 0.92)**	**<0.01**	0.83 (0.67, 1.02)	0.070

*BMI, body mass index; GS, grip strength; PA, physical activity; METs, metabolic equivalents. ^*a*^Obtained by using multiple logistic regression analysis. ^*b*^Additionally adjusted for education level, income status, marriage status, employment status. ^*c*^Additionally adjusted for education level, income status, marriage status, employment status, physical activity, smoking status, drinking status, sleep duration. ^*d*^Additionally adjusted for education level, income status, marriage status, employment status, physical activity, smoking status, drinking status, sleep duration, BMI, GS, hypertension, diabetes, hyperlipidemia, sleepiness. ^*e*^Boldface indicates statistical significance (*P* < 0.05) (or appropriate value).*

## Discussion

We observed that the age- and sex-standardized prevalence of MCI in people living in Northern China was 10.7%. Also, our results revealed that certain sociodemographic and health-related characteristics were associated with the prevalence of MCI. This study may have implications for the development of MCI prevention and healthy aging policy.

The prevalence of MCI observed in this study was similar (10.7%) to that previously reported in other regions of China (9.7–16.5%) (Nie et al., 2011). Consistent with the sex-specific prevalence shown in a meta-analysis from China (Nie et al., 2011), the current study demonstrated a higher prevalence of MCI in females than in males. In addition, the overall pattern of MCI prevalence across different age groups in this study was similar to the results of a meta-analysis (Nie et al., 2011), where the MCI prevalence was much higher and continued to increase after 70 years of age, suggesting that age-related changes likely play a greater role in MCI after 70 years. Although many studies calculated the prevalence of MCI, they were limited in terms of having a small sample size, limited number of risk factors assessed, and lack of stratified analysis. Therefore, more extensive and in-depth analyses of the association between risk factors and the prevalence of MCI are warranted.

Earlier studies found an association between MCI and sociodemographic characteristics, including age, sex, education level, income status, and marital status in older adults (Rawtaer et al., 2017; Vanoh et al., 2017; Tsoy et al., 2019; Fernandez-Blazquez et al., 2020). However, we found that most of the recognized risk factors for MCI were not different between those with and without MCI. However, employment status in the youngest group and age distribution were significantly different between those with and without MCI in the sample analyzed. There are inconsistencies between studies regarding the association between age and MCI (Vanoh et al., 2017; Hussin et al., 2019; Kume et al., 2019). In this analysis, we excluded participants with cerebrovascular disease, AD, and severe limitations in ADL, leading to fewer MCI cases in the 65–69 age group. This may explain our observations that participants in the second age stratum (65–69 years) had a significantly lower prevalence of MCI when compared to people aged 60–64 years. In addition, the results of this study also showed that the higher age group (≥70 years) was associated with lower MMSE scores regardless of their ADL scores. It has been suggested that the prevalence of MCI was significantly lower for those aged less than 70 years, demonstrating an association between age and MCI in this study. In the sensitivity analysis, the oldest age group and females were found to be associated with an increased prevalence of MCI. However, the association disappeared after adjusting for the proximal variables, which indicated that proximal factors might mediate the association of age and sex with MCI. Therefore, the proximal factors (i.e., BMI, GS, sleepiness, hypertension, diabetes, and hyperlipidemia) were considered as modifiable factors that can be targeted to reduce the prevalence of MCI. Few studies have focused on the association between employment status in people aged over 60 years and MCI (Mohan et al., 2019). Going to work results in more opportunities to go out and participate in social activities that are potential protective factors for MCI (Anderson et al., 2012; Gao et al., 2018). The employment status of Chinese over 60 years of age gradually changes after retirement. Similarly, in this study, as age increased, the number of adults having jobs gradually declined. Almost all of the participants in the oldest age group were retired. This could explain why the working status in the youngest group was positively associated with cognitive function in this study. Although no association was observed between other sociodemographic variables and the prevalence of MCI, these variables, including sex, education level, income status, and marital status, were associated with cognitive function scores. Therefore, these variables may be essential in informing strategies to improve cognitive function.

We found that participants with lower levels of PA, BMI, and GS, higher levels of sleepiness, longer sleep duration, and hypertension were associated with a higher prevalence of MCI. These findings are in accordance with previous studies (Babkoff et al., 1991; Sofi et al., 2011; Keage et al., 2012; Roberts and Knopman, 2013; Beydoun et al., 2014; Pearson et al., 2016; Liang et al., 2019; Liu et al., 2020; Pacifico et al., 2020). However, participants with MCI tended to be non-drinkers. This may contradict previous research findings (Mira et al., 2019). One possible reason is that participants with MCI who tended to be older and have hypertension may, in turn, change their lifestyle (e.g., quit drinking). Therefore, after multivariate adjustments, the association between drinking status and MCI disappeared. Besides, several meta-analyses have found that low to moderate alcohol drinking is associated with better global cognition scores, whereas excessive alcohol intake elevates the risk of progression to dementia in people with MCI (Lao et al., 2020; Zhang et al., 2020). However, we did not investigate alcohol consumption among those with and without MCI. Further studies on the association between alcohol consumption and MCI is required.

In this study, a negative association between BMI as either a categorical or a continuous variable and MCI was observed. Moreover, the inverse association was strengthened with increased age, and BMI was also positively associated with cognitive function scores. Similarly, other cross-sectional studies reported that being overweight was linked to a decreased prevalence of cognitive impairment in Chinese and Indonesian elderly (Hou et al., 2019; Vidyanti et al., 2020). In contrast, several prospective studies that analyzed the effects of BMI trajectories from middle to old age on cognitive function showed that deceleration of weight gain at older ages reflected early signs of cognitive impairment (Wagner et al., 2020; Bohn et al., 2020). However, elevated body weight in middle-age might reduce cognitive function (Singh-Manoux et al., 2012; Suemoto et al., 2015; Wagner et al., 2020; Bohn et al., 2020; Floud et al., 2020). A recent systematic review is in agreement with our findings that AD risk is decreased when BMI surpassed 27 kg/m^2^ in later life, suggesting that the elderly could increase their body weight to combat dementia (Qu et al., 2020). Several biologic processes have explained that higher BMI in later life may be beneficial by increasing insulin-like growth factor I (IGF-1) levels as well as leptin hormone levels and estrogen production (Yamamoto and Kato, 1993; Harvey et al., 2006; Singh et al., 2006), all of which are associated with better cognitive performance (Power et al., 2011). Furthermore, after age stratification, it was found that the negative association between BMI and the prevalence of MCI was strengthened with age. Although specific mechanisms for this observation remain unclear, it could be related to factors in specific settings. For example, the aging process involves multiple psychosocial, behavioral, and physiological changes, which may partially explain the differential associations between BMI and MCI across different age stages. More in-depth studies are warranted to explain the association between higher BMI and cognitive function in later life.

The association between hyperlipidemia and the prevalence of MCI remains controversial (Xue et al., 2017; Chen et al., 2018). A review and meta-analysis revealed that midlife high total serum cholesterol was associated with an increased risk of MCI, AD, and cognitive decline in later life. However, high cholesterol in later life was not associated with MCI, AD, dementia, or cognitive decline (Anstey et al., 2017). Furthermore, similar to previous studies, our study found a non-significant negative association between hyperlipidemia and the prevalence of MCI in the elderly, although the association was further strengthened in the sensitivity analysis. Interestingly, when stratified according to sex, the associations between serum lipids and cognitive impairment were only prominent in older females. Similarly, in a study by Kim and Park (2017), hyperlipidemia was reported as a protective factor for MCI in females. Furthermore, among the four indicators causing hyperlipidemia (i.e., TC, TG, LDL-C, and HDL-C), only TG was related to the prevalence of MCI among older females. Moreover, there appeared to be heterogeneity in the association between TG and MCI by sex. However, only a few studies have analyzed the relationship between serum lipids and cognitive impairment, depending on sex. In contrast, a cross-sectional study in rural China on serum lipids revealed an inverse association between TG and the prevalence of MCI in middle-aged males and a positive association between LDL-C and MCI in older females (Zhao et al., 2019). The discrepancy between that study and our findings may be partially due to differences in sample size, dietary habits, and MCI diagnostic criteria. The mechanisms by which increased TG improves cognitive function could be due to the following: Low TG concentrations have been suggested to be correlated with brain inflammation, frailty, low nutrition levels, and low endogenous estrogen (Hu et al., 2003; Yasui et al., 2008). Estrogen may facilitate better cognitive function by exerting effects on specific brain regions such as the prefrontal cortex and hippocampus (Hara et al., 2015). This may explain the positive association between TG levels and cognitive function only in females. On the other hand, low TG levels may, in turn, reflect a low nutrition level implying pathological changes or be a marker for early cognitive impairment. However, potential mechanisms underlying the observed interactions between sex and blood lipid indicators with cognitive impairment are still unclear. Moreover, the reasons for this inconsistency in different sexes could partly be because cognitive impairment has quite distinct sex differences in terms of innate physiology, social behavior, and relevant factors (Ritchie et al., 2010). As this is a cross-sectional study, further prospective research is needed to explore the sex differences with the association of hyperlipidemia and its diagnostic indicators with MCI.

The ratio of males to females (1:1.26) and the education level among the Northern Chinese sample are in line with the averages in China as a whole (Zhang, 2016; Zeng and Zhao, 2019). The prevalence rate differences between males and females observed in this study align well with the data from other parts of China (Xue et al., 2018). Due to the acceleration of urbanization, improvement of living standards, and the heavy-flavored diet of the Northern population (i.e., due to excessive use of salt) (Fang et al., 2020), the Northern Chinese have a higher prevalence of hypertension compared to those in the South (Zhao et al., 2004). As the survey site is limited to the Northern region, the findings of the current study can only be generalized to Northern (Chinese) older adults.

Our study is among the first to explore the sex- and age-specific prevalence of MCI among adults aged 60 years and over in Northern China, and to analyze an extensive list of risk factors for MCI. Moreover, we used rigorous and standardized protocols and quality control procedures for data collection and adjusted for the age and sex structure of populations in the prevalence estimates to enable comparisons with other studies. However, this study has several limitations. Due to the cross-sectional nature of this study, the observed associations may be influenced by reverse causation, particularly for lifestyle-related factors. However, we performed several sensitivity analyses to evaluate the potential for reverse causation, and the results remained unchanged. Next, although numerous sociodemographic and health-related factors were retained in the final models, residual confounding may still exist. Furthermore, since we did not have data on estrogen levels, we were unable to explore potential mechanisms underlying the association between TG and cognitive function in older females. Therefore, future studies should measure estrogen to examine the reasons for the association between TG and cognitive function in older females more fully. Finally, since this study was a field survey, elderly adults with limited mobility were not included. Therefore, further household surveys are needed to yield more generalizable findings.

In conclusion, 10.7% of all adults aged 60 years and above were found to have MCI in this study. The prevalence of MCI was higher in females and older age groups. In addition, PA, BMI, and GS were inversely associated with MCI, whereas sleepiness, longer sleep duration, and hypertension tended to increase the prevalence of MCI. TG and BMI might have different associations with the presence of MCI at different sex and age stages, respectively. Our data highlight the need for mechanism studies to better understand differences in the associations between multiple influencing factors and MCI. Moreover, large prospective studies with detailed baseline data, follow-up of health-related factors, and cognitive impairment-related outcomes are required so that individual risk can be predicted and managed by calculating risk scores for cognitive impairment. Therefore, further prospective studies or clinical trials are required.

## Data Availability Statement

The raw data supporting the conclusions of this article will be made available by the authors, without undue reservation.

## Ethics Statement

The studies involving human participants were reviewed and approved by the Institutional Review Board of Tianjin Medical University. The patients/participants provided their written informed consent to participate in this study. Written informed consent was obtained from the individual(s) for the publication of any potentially identifiable images or data included in this article.

## Author Contributions

JF contributed to the statistical analysis, interpretation of the data, and drafting of the manuscript. JF, QL, YD, CS, HL, and MJ contributed to the acquisition of the data. YD, YZ, FM, WL, HL, XZ, YC, ZS, GW, and GH contributed to the conception, design, and revision of the manuscript. JF, QL, HL, and MJ contributed to the assembly of the data. JF, QL, YD, GW, and GH contributed to the approval of the final version of the manuscript. All the authors contributed to the article and approved the submitted version.

## Conflict of Interest

The authors declare that the research was conducted in the absence of any commercial or financial relationships that could be construed as a potential conflict of interest.
