# Supplementary material for: Age- and Sex-Specific Prevalence and Modifiable Risk Factors of Mild Cognitive Impairment Among Older Adults in China: A Population-Based Observational Study
Source: Front Aging Neurosci. 2020 Oct 30;12:578742. doi: 10.3389/fnagi.2020.578742 (PMC7662098; doi:10.3389/fnagi.2020.578742)
Supplement: Supplementary file 1 [file Table_1.DOCX]

**Supplementary Online Content**

**Supplementary Table 1. Characteristics of the participants with and without completed questionnaire information (n=4,766)**

**Supplementary Table 2. Associations between the risk factors and MCI after excluding participants who had long-term medication use (adjusted ORs and 95 % CIs; n=3,833)**

**Supplementary Table 3. Associations between the risk factors and MCI (adjusted ORs and 95 % CIs; n=4,766)**

**Supplementary Table 4. Associations between the risk factors and MCI after stratified by sex (adjusted ORs and 95 % CIs)**

**Supplementary Table 5. Associations between the risk factors and MCI after stratified by age (adjusted ORs and 95 % CIs)**

| **Supplementary Table 1. Characteristics of the participants with and without completed questionnaire information (n=4,766)** | | | |
| --- | --- | --- | --- |
| **Characteristics** | **Completed questionnaire information** | | ***P* value** ^a^ |
|  | **No** | **Yes** |  |
| **No. of subjects** | 135 | 4,631 |  |
| **Sociodemographic characteristics** | |  |  |
| **Age group (years)** | 64.5 (63.7, 65.4) | 67.4 (67.3, 67.5) | < 0.0001 |
| **Sex (Males, %)** | 39.0 | 44.3 | 0.22 |
| **High school and above (%)** | 6.62 | 16.4 | < 0.01 |
| MCI mild cognitive impairment.  ^a^ Analysis of logistic regression analysis. | | | |

| **Supplementary Table 2. Associations between the risk factors and MCI after excluding participants who had long-term medication use (adjusted ORs and 95 % CIs; n=3,833)** | | | | | |
| --- | --- | --- | --- | --- | --- |
| **Variables** | **Age and sex adjusted** | |  | **Multivariable adjusted** ^b^ | |
|  | **OR (95%CI)** | ***P* Value** ^a^ |  | **OR (95%CI)** | ***P* Value** |
| **Sociodemographic characteristics** | |  |  |  |  |
| **Age group (years)** |  |  |  |  |  |
| 60-64 | 1.00 (reference) | - |  | 1.00 (reference) | - |
| 65-69 | 0.85 (0.65, 1.12) | 0.24 |  | **0.76 (0.57, 1.00)** | **0.048** |
| 70-74 | 1.12 (0.84, 1.50) | 0.45 |  | 0.88 (0.64, 1.19) | 0.40 |
| ≥75 | **1.88 (1.34, 2.62)** | **<0.001** |  | 1.39 (0.97, 1.98) | 0.073 |
| ***P* for trend** ^a^ | **<0.01** |  |  | 0.31 |  |
| **Sex (Females vs Males)** | **1.61 (1.29, 2.01)** | **<0.0001** |  | 1.11 (0.81, 1.54) | 0.52 |
| **High school and above** | **0.64 (0.45, 0.89)** | **0.011** |  | 0.73 (0.50, 1.04) | 0.093 |
| **Income status (RMB)** |  |  |  |  |  |
| < 3, 000 | 1.00 (reference) | - |  | 1.00 (reference) | - |
| 3, 000-5, 000 | 1.00 (0.73, 1.35) | 0.99 |  | 1.30 (0.93, 1.78) | 0.11 |
| >5, 000 | 0.74 (0.51, 1.04) | 0.092 |  | 1.07 (0.72, 1.56) | 0.73 |
| ***P* for trend** | 0.14 |  |  | 0.90 |  |
| **Married vs Unmarried** | 0.80 (0.59, 1.08) | 0.14 |  | 0.86 (0.63, 1.18) | 0.33 |
| **Working vs No work** | 0.84 (0.49, 1.36) | 0.50 |  | 0.79 (0.46, 1.29) | 0.37 |
| **Health related variables** |  |  |  |  |  |
| **PA (METs × h/w) (**≥23.0 vs < 23.0**)**^c^ | **0.70 (0.56, 0.87)** | **< 0.01** |  | **0.74 (0.59, 0.92)** | **< 0.01** |
| **Smoking status** |  |  |  |  |  |
| Non-smoker | 1.00 (reference) | - |  | 1.00 (reference) | - |
| Ex-smoker | 0.57 (0.31, 0.98) | 0.058 |  | 0.55 (0.30, 0.95) | **0.045** |
| Current smoker | 1.19 (0.91, 1.55) | 0.21 |  | 1.20 (0.90, 1.57) | 0.21 |
| **Supplementary Table 2. Associations between the risk factors and MCI after excluding participants who had long-term medication use (adjusted ORs and 95 % CIs; n=3,833)** | | | | | |
| **Variables** | **Age and sex adjusted** | |  | **Multivariable adjusted** ^b^ | |
|  | **OR (95%CI)** | ***P* Value** ^a^ |  | **OR (95%CI)** | ***P* Value** |
| ***P* for trend** | 0.22 |  |  | 0.17 |  |
| **Alcohol drinking** | 0.88 (0.64, 1.23) | 0.46 |  | 0.87 (0.62, 1.21) | 0.41 |
| **Sleep duration (h)** |  |  |  |  |  |
| < 6.5 h | 0.95 (0.56, 1.52) | 0.85 |  | 0.93 (0.55, 1.50) | 0.77 |
| 6.5-8.5 h | 1.00 (reference) | - |  | 1.00 (reference) | - |
| > 8.5 h | **1.42 (1.14, 1.77)** | **<0.01** |  | **1.29 (1.02, 1.62)** | **0.030** |
| ***P* for trend** | **<0.01** |  |  | **<0.01** |  |
| **Sleepiness (scores)** | **1.80 (1.32, 2.44)** | **<0.001** |  | **1.74 (1.27, 2.37)** | **<0.001** |
| **BMI (kg/m^2^)** | **0.94 (0.91, 0.98)** | **<0.001** |  | **0.92 (0.89, 0.95)** | **<0.0001** |
| **GS (per body weight) (kg/kg)** | **0.56 (0.41, 0.76)** | **<0.001** |  | **0.50 (0.36, 0.69)** | **<0.0001** |
| **Hypertension** | **1.35 (1.09, 1.68)** | **<0.01** |  | **1.46 (1.16, 1.83)** | **<0.01** |
| **Diabetes** | 1.00 (0.75, 1.32) | 1.00 |  | 0.99 (0.73, 1.31) | 0.92 |
| **Hyperlipidemia** | **0.72 (0.58, 0.90)** | **<0.01** |  | **0.79 (0.63, 1.00)** | **0.045** |
| BMI body mass index, GS grip strength, PA physical activity, METs metabolic equivalents.  ^a^ Obtained by using multiple logistic regression analysis.  ^b^ Additionally adjusted for education level, income status, marriage status, employment status, physical activity, smoking status, drinking status, sleep duration, BMI, GS, hypertension, diabetes, hyperlipidemia, sleepiness.  ^c^ Boldface indicates statistical significance (*P*< 0.05) (or appropriate value). | | | | | |

| **Supplementary Table 3. Associations between the risk factors and MCI (adjusted ORs and 95 % CIs; n=4,766)** | | | | | |
| --- | --- | --- | --- | --- | --- |
| **Variables** | **Age and sex adjusted** | |  | **Multivariable adjusted** ^b^ | |
|  | **OR (95%CI)** | ***P* Value** ^a^ |  | **OR (95%CI)** | ***P* Value** |
| **Sociodemographic characteristics** | |  |  |  |  |
| **Age group (years)** |  |  |  |  |  |
| 60-64 | 1.00 (reference) | - |  | 1.00 (reference) | - |
| 65-69 | 0.80 (0.62, 1.02) | 0.067 |  | **0.72 (0.56, 0.93)** | **0.010** |
| 70-74 | 1.25 (0.98, 1.61) | 0.075 |  | 0.98 (0.75, 1.27) | 0.86 |
| ≥75 | **1.60 (1.17, 2.15)** | **<0.0001** |  | 1.16 (0.84, 1.60) | 0.36 |
| ***P* for trend** ^a^ | **<0.001** |  |  | 0.15 |  |
| **Females vs Males** | **1.67 (1.37, 2.03)** | **<0.0001** |  | 1.13 (0.85, 1.51) | 0.39 |
| **High school and above** | **0.66 (0.48, 0.89)** | **<0.01** |  | 0.79 (0.57, 1.11) | 0.18 |
| **Income status (RMB)** |  |  |  |  |  |
| < 3, 000 | 1.00 (reference) | - |  | 1.00 (reference) | - |
| 3, 000-5, 000 | 0.86 (0.64, 1.14) | 0.30 |  | 1.08 (0.80, 1.45) | 0.61 |
| >5, 000 | **0.68 (0.48, 0.93)** | **0.019** |  | 0.92 (0.65, 1.31) | 0.64 |
| ***P* for trend** | **0.014** |  |  | 0.71 |  |
| **Married vs Unmarried** ^c^ | 0.81 (0.63, 1.07) | 0.13 |  | 0.88 (0.67, 1.16) | 0.36 |
| **Working vs No work** ^c^ | 0.70 (0.43, 1.10) | 0.14 |  | 0.68 (0.42, 1.10) | 0.12 |
| **Health related variables** |  |  |  |  |  |
| **PA (METs × h/w) (**≥23.0 vs < 23.0**)^c^** | **0.73 (0.60, 0.88)** | **<0.01** |  | **0.78 (0.64, 0.95)** | **0.015** |
| **Smoking status** |  |  |  |  |  |
| Non-smoker | 1.00 (reference) | - |  | 1.00 (reference) | - |
| Ex-smoker | 0.75 (0.47, 1.17) | 0.23 |  | 0.70 (0.44, 1.11) | 0.13 |
| Current smoker | 1.20 (0.94, 1.52) | 0.13 |  | 1.20 (0.94, 1.55) | 0.14 |
| ***P* for trend** | 0.14 |  |  | 0.16 |  |
| **Supplementary Table 3. Associations between the risk factors and MCI (adjusted ORs and 95 % CIs; n=4,766)** | | | | | |
| **Variables** | **Age and sex adjusted** | |  | **Multivariable adjusted** ^b^ | |
|  | **OR (95%CI)** | ***P* Value** ^a^ |  | **OR (95%CI)** | ***P* Value** |
| **Alcohol drinking** | 0.84 (0.62, 1.12) | 0.24 |  | 1.22 (0.90, 1.65) | 0.21 |
| **Sleep duration (h)** |  |  |  |  |  |
| < 6.5 h | 0.96 (0.60, 1.46) | 0.85 |  | 0.94 (0.60, 1.47) | 0.77 |
| 6.5-8.5 h | 1.00 (reference) | - |  | 1.00 (reference) | - |
| > 8.5 h | **1.55 (1.28, 1.89)** | **<0.0001** |  | **1.38 (1.13, 1.69)** | **<0.01** |
| ***P* for trend** | **<0.001** |  |  | **<0.0001** |  |
| **Sleepy state (scores)** | **1.80 (1.37, 2.36)** | **<0.0001** |  | **1.72 (1.31, 2.27)** | **<0.001** |
| **BMI (kg/m^2^)** | **0.95 (0.92, 0.98)** | **<0.001** |  | **0.93 (0.90, 0.96)** | **<0.0001** |
| **GS (per body weight) (kg/kg)** | **0.54 (0.41, 0.71)** | **<0.0001** |  | **0.51 (0.38, 0.67)** | **<0.0001** |
| **Hypertension** | **1.32 (1.09, 1.60)** | **<0.01** |  | **1.40 (1.15, 1.72)** | **<0.01** |
| **Diabetes** | 1.00 (0.78, 1.27) | 0.98 |  | 0.99 (0.77, 1.28) | 0.96 |
| **Hyperlipidemia** | **0.73 (0.60, 0.88)** | **<0.01** |  | 0.83 (0.67, 1.02) | 0.080 |
| BMI body mass index, GS grip strength, PA physical activity, METs metabolic equivalents.  ^a^ Obtained by using multiple logistic regression analysis.  ^b^ Additionally adjusted for education level, income status, marriage status, employment status, physical activity, smoking status, drinking status, sleep duration, BMI, GS, hypertension, diabetes, hyperlipidemia, sleepy state.  ^c^ Boldface indicates statistical significance *(P*< 0.05) (or appropriate value). | | | | | |

| **Supplementary Table 4. Associations between the risk factors and MCI after stratified by sex (adjusted ORs and 95 % CIs)** | | | | | | | | | | | |
| --- | --- | --- | --- | --- | --- | --- | --- | --- | --- | --- | --- |
| **Variables** | **Age adjusted** | | | | | ***P* for heterogeneity** ^c^ | **Multivariable adjusted** ^b^ | | | | ***P* for heterogeneity** |
|  | **Males (n=2,052)** | | | **Females (n=2,579)** | |  | **Males (n=2,052)** | | **Females (n=2,579)** | |  |
|  | **OR (95%CI)** | | ***P* Value** ^a^ | **OR (95%CI)** | ***P* Value** |  | **OR (95%CI)** | ***P* Value** | **OR (95%CI)** | ***P* Value** |  |
| **Sociodemographic characteristics** | | |  |  |  |  |  |  |  |  |  |
| **Age group (years)**^e^ |  | |  |  |  |  |  |  |  |  | **-** |
| 60-64 | 1.00 (reference) | | - | 1.00 (reference) | - | - | 1.00 (reference) | - | 1.00 (reference) | - | - |
| 65-69 | 0.74(0.49, 1.12) | | 0.15 | 0.87(0.63, 1.19) | 0.41 | 0.51 | 0.68(0.44, 1.04) | 0.073 | 0.80(0.58, 1.09) | 0.16 | 0.55 |
| 70-74 | 1.05(0.69, 1.60) | | 0.83 | **1.47(1.06, 2.03)** | **0.024** | 0.24 | 0.86(0.54, 1.34) | 0.50 | 1.14(0.82, 1.59) | 0.45 | 0.32 |
| ≥75 | 1.20(0.70, 1.99) | | 0.49 | **2.01(1.33, 2.98)** | **<0.001** | 0.11 | 0.95(0.53, 1.64) | 0.85 | 1.49(0.97, 2.25) | 0.063 | 0.21 |
| ***P* for trend** ^a^ | 0.39 | | - | **<0.0001** | - | - | 0.85 | - | **0.048** | - | - |
| **High school and above** | 0.81(0.54, 1.19) | | 0.30 | **0.51(0.29, 0.84)** | **0.012** | 0.13 | 0.95(0.61, 1.46) | 0.82 | 0.64(0.36, 1.09) | 0.12 | 0.28 |
| **Income status (RMB)** |  | |  |  |  |  |  |  |  |  |  |
| < 3,000 | 1.00 (reference) | | - | 1.00 (reference) | - | - | 1.00 (reference) | - | 1.00 (reference) | - | - |
| 3,000-5,000 | 1.11(0.71, 1.68) | | 0.63 | 0.76(0.51, 1.10) | 0.16 | 0.17 | 1.31(0.82, 2.04) | 0.24 | 0.97(0.64, 1.43) | 0.87 | 0.32 |
| >5,000 | 0.67 (0.40, 1.07) | | 0.11 | 0.71(0.44, 1.07) | 0.12 | 0.94 | 0.86(0.49, 1.47) | 0.60 | 0.99(0.61, 1.56) | 0.97 | 0.71 |
| ***P* for trend** | 0.19 | | - | 0.055 | - | - | 0.92 | - | 0.92 | - | - |
| **Married vs Unmarried** | **0.59(0.38, 0.94)** | | **0.022** | 0.96(0.69, 1.36) | 0.81 | 0.16 | 0.63(0.40, 1.02) | 0.049 | 1.05(0.74, 1.50) | 0.80 | 0.084 |
| **Working vs No work** | 0.85 (0.47, 1.45) | | 0.57 | 0.41(0.13, 1.00) | 0.086 | 0.16 | 0.80(0.43, 1.38) | 0.44 | 0.42(0.13, 1.03) | 0.096 | 0.28 |
| **Health related variables** | | |  |  |  |  |  |  |  |  |  |
| **PA (METs × h/w)** ^d^ | **0.68(0.49, 0.94)** | | **0.019** | **0.75(0.59, 0.96)** | **0.020** | 0.69 | 0.74(0.53, 1.04) | 0.085 | 0.82(0.64, 1.05) | 0.12 | 0.66 |
| **Smoking status** |  | |  |  |  |  |  |  |  |  |  |
| Non-smoker | 1.00 (reference) | | - | 1.00 (reference) | - |  | 1.00 (reference) | - | 1.00 (reference) | - |  |
| Ex-smoker | 0.70(0.39, 1.22) | | 0.23 | 0.75(0.26, 1.73) | 0.54 | 0.91 | 0.63(0.34, 1.10) | 0.12 | 0.75(0.25, 1.74) | 0.54 | 0.76 |
| **Supplementary Table 4. Associations between the risk factors and MCI after stratified by sex (adjusted ORs and 95 % CIs)** | | | | | | | | | | | |
| **Variables** | **Age adjusted** | | | | | ***P* for heterogeneity** ^c^ | **Multivariable adjusted** ^b^ | | | | ***P* for heterogeneity** |
|  | **Men (n=2,052)** | | | **Women (n=2,579)** | |  | **Men (n=2,052)** | | **Women (n=2,579)** | |  |
|  | **OR (95%CI)** | ***P* Value** ^a^ | | **OR (95%CI)** | ***P* Value** |  | **OR (95%CI)** | ***P* Value** | **OR (95%CI)** | ***P* Value** |  |
| Current smoker | 1.26(0.89, 1.80) | | 0.19 | 1.09(0.76, 1.54) | 0.62 | 0.51 | 1.28(0.89, 1.85) | 0.19 | 1.07(0.74, 1.53) | 0.88 | 0.51 |
| ***P* for trend** | 0.16 |  | | 0.69 |  |  | 0.16 |  | 0.78 |  |  |
| **Alcohol drinking** | 0.83(0.60, 1.15) | 0.26 | | 0.93(0.41, 1.86) | 0.85 | 0.84 | 0.81(0.57, 1.30) | 0.22 | 0.94(0.40, 1.93) | 0.87 | 0.73 |
| **Sleep duration (h)** |  |  | |  |  |  |  |  |  |  |  |
| < 6.5 h | 1.03(0.47, 2.00) | 0.94 | | 0.94(0.50, 1.62) | 0.82 | 0.34 | 0.96(0.43, 1.92) | 0.97 | 0.96(0.51, 1.68) | 0.88 | 0.95 |
| 6.5-8.5 h | 1.00 (reference) | - | | 1.00 (reference) | - | - | 1.00 (reference) | - | 1.00 (reference) | - |  |
| > 8.5 h | 1.40(1.00, 1.96) | 0.051 | | **1.66(1.30, 2.14)** | **<0.0001** | **0.046** | 1.19(0.83, 1.13) | 0.30 | **1.51(1.17, 1.96)** | **<0.01** | 0.59 |
| ***P* for trend** | 0.14 |  | | **<0.001** |  |  | 0.40 |  | **<0.01** |  |  |
| **Sleepiness (scores)** | **2.48(1.56, 3.91)** | **<0.0001** | | **1.57(1.11, 2.22)** | **<0.01** | 0.11 | **2.50(1.56, 3.99)** | **<0.001** | **1.49(1.04, 2.11)** | **0.026** | 0.083 |
| **BMI (kg/m^2^)** | 0.97(0.91, 1.02) | 0.20 | | **0.94(0.91, 0.97)** | **<0.001** | 0.34 | **0.94 (0.88, 0.99)** | **0.028** | **0.92(0.88, 0.95)** | **<0.0001** | 0.50 |
| **GS (per body weight) (kg/kg)** | **0.56(0.35, 0.91)** | **0.018** | | **0.52(0.37, 0.73)** | **<0.001** | 0.57 | **0.60(0.36, 0.99)** | **0.047** | **0.47(0.33, 0.66)** | **<0.0001** | 0.42 |
| **Hypertension** | **1.68(1.21, 2.35)** | **<0.01** | | 1.21(0.94, 1.54) | 0.14 | 0.14 | **1.68(1.19, 2.38)** | **<0.01** | **1.32(1.02, 1.70)** | **0.038** | 0.27 |
| **Diabetes** | 1.11(0.72, 1.65) | 0.64 | | 1.00(0.73, 1.35) | 0.99 | 0.73 | 1.01(0.64, 1.54) | 0.98 | 0.99(0.72, 1.35) | 0.97 | 0.96 |
| **Hyperlipidemia** | 1.00(0.73, 1.39) | 0.99 | | **0.63(0.49, 0.81)** | **<0.001** | **0.020** | 1.04(0.74, 1.47) | 0.81 | **0.70(0.54, 0.91)** | **<0.01** | 0.066 |
| **TC (mmol/L)** | 1.11(0.54, 2.38) | 0.78 | | 0.81(0.47, 1.43) | 0.45 | 0.49 | 1.25(0.57, 2.77) | 0.59 | 1.13 (0.63, 2.06) | 0.69 | 0.84 |
| **TG (mmol/L)** | 1.10 (0.79, 1.50) | 0.57 | | **0.60(0.46, 0.78)** | **<0.001** | **<0.01** | 1.14(0.80, 1.63) | 0.46 | **0.66(0.50, 0.87)** | **<0.01** | **0.016** |
| **LDL-C (mmol/L)** | 1.52(0.84, 2.80) | 0.17 | | 1.22(0.78, 1.93) | 0.39 | 0.56 | 1.56(0.80, 3.09) | 0.21 | 1.43(0.90, 2.31) | 0.13 | 0.94 |
| **HDL-C (mmol/L)** | 0.80(0.42, 1.41) | 0.48 | | 1.48(0.85, 2.59) | 0.17 | 0.15 | 0.77(0.39, 1.46) | 0.48 | 1.35(0.75, 2.42) | 0.32 | 0.24 |
| BMI body mass index, GS grip strength, PA physical activity, METs metabolic equivalents, TG triglyceride;  ^a^ Obtained by using multiple logistic regression analysis.  ^b^ Additionally adjusted for education level, income status, marriage status, employment status, physical activity, smoking status, drinking status, sleep duration, BMI, GS, hypertension, diabetes, hyperlipidemia or its diagnostic indicators, sleepy state.  ^c^ Reflects comparison between odds ratio associated with man vs women using the statistical of heterogeneity.  ^d^ Physical activity (≥23.0 vs < 23.0).  ^e^. unadjusted other variables.  ^f^ Boldface indicates statistical significance (*P*< 0.05) (or appropriate value). | | | | | | | | | | | |

| **Supplementary Table 5. Associations between the risk factors and MCI after stratified by age (adjusted ORs and 95 % CIs)** | | | | | | | | | | |
| --- | --- | --- | --- | --- | --- | --- | --- | --- | --- | --- |
| **Variables** | **Sex adjusted** | | | | ***P* for heterogeneity**^c^ | **Multivariable adjusted** ^b^ | | | | ***P* for heterogeneity** |
|  | **60-64 (n=1,464)** | **65-69 (n=1,633)** | **70-74**  **(n=1,069)** | ≥**75**  **(n=465)** |  | **60-64 (n=1,464)** | **65-69 (n=1,633)** | **70-74**  **(n=1,069)** | ≥**75**  **(n=465)** |  |
| **Sociodemographic characteristics** | |  |  |  |  |  |  |  |  |  |
| **Females vs Males** ^f,g,h,m^ | 1.30  (0.91, 1.88) | **1.55**  **(1.07, 2.28)** | **1.79**  **(1.22, 2.67)** | **2.22**  **(1.31, 3.86)** | 0.39 | 0.90  (0.52, 1.58) | 1.17  (0.68, 2.05) | 1.17  (0.67, 2.06) | 1.41  (0.66, 3.08) | 0.83 |
| **High school and above** ^g^ | 0.64  (0.38, 1.02) | 1.05  (0.60, 1.75) | **0.46 (0.19, 0.95)** | 0.49  (0.17, 1.19) | 0.23 | 0.79  (0.45, 1.33) | 1.06  (0.58, 1.86) | 0.80  (0.31, 1.84) | 0.58  (0.18, 1.56) | 0.75 |
| **Income status (RMB)** |  |  |  |  |  |  |  |  |  |  |
| < 3,000 | 1.00 (reference) | 1.00 (reference) | 1.00  (reference) | 1.00 (reference) | - | 1.00 (reference) | 1.00 (reference) | 1.00 (reference) | 1.00  (reference) | - |
| 3,000-5,000 | 1.03  (0.63, 1.64) | 1.26  (0.75, 2.04) | **0.55**  **(0.28, 0.98)** | 0.62  (0.21, 1.51) | 0.16 | 1.33  (0.79, 2.16) | 1.44  (0.84, 2.37) | 0.79  (0.39, 1.48) | 0.80  (0.25, 2.11) | 0.45 |
| >5,000 ^e, g^ | **0.52**  **(0.27, 0.92)** | 1.27  (0.73, 2.10) | **0.32**  **(0.12, 0.69)** | 0.80  (0.29, 1.86) | **0.025** | 0.72  (0.35, 1.37) | 1.50  (0.84, 2.59) | 0.48  (0.17, 1.13) | 1.56  (0.51, 4.23) | 0.11 |
| ***P* for trend** | 0.069 | 0.25 | **<0.01** | 0.43 |  | 0.68 | 0.094 | 0.10 | 0.58 | - |
| **Married vs Unmarried** ^g^ | 0.80  (0.47, 1.47) | 1.38  (0.74, 2.85) | **0.61**  **(0.39, 0.99)** | 0.79  (0.45, 1.41) | 0.27 | 0.84  (0.48, 1.55) | 1.47  (0.78, 3.08) | 0.62  (0.39, 1.03) | 0.90  (0.49, 1.69) | 0.25 |
| **Working vs No work** ^e,i^ | **0.43**  **(0.18, 0.88)** | 1.43  (0.70, 2.65) | 0.31  (0.02, 1.51) | - | 0.13 | **0.40**  **(0.17, 0.84)** | 1.57  (0.76, 2.98) | 0.30  (0.02, 1.54) | - | 0.060 |
| **Health related variables** |  |  |  |  |  |  |  |  |  |  |
| **PA (METs × h/w)** ^d, g, k^ | 0.70  (0.49, 1.01) | 0.79  (0.55, 1.15) | **0.58**  **(0.40, 0.85)** | 0.95  (0.57, 1.62) | 0.47 | 0.76  (0.53, 1.10) | 0.83  (0.57, 1.22) | **0.67**  **(0.46, 1.00)** | 0.96  (0.55, 1.72) | 0.80 |
| **Smoking status** |  |  |  |  |  |  |  |  |  |  |
| Non-smoker | 1.00 (reference) | 1.00 (reference) | 1.00 (reference) | 1.00 (reference) | - | 1.00 (reference) | 1.00 (reference) | 1.00 (reference) | 1.00 (reference) | - |
| Ex-smoker | 0.89  (0.35, 1.97) | 0.86  (0.35, 1.87) | 0.63  (0.23, 1.43) | 0.20  (0.01, 1.00) | 0.41 | 0.80  (0.31, 1.82) | 0.90  (0.36, 1.98) | 0.52  (0.19, 1.22) | 0.19  (0.01, 1.00) | 0.49 |
| Smoker | 1.15  (0.73, 1.80) | 1.28  (0.81, 2.00) | 1.08  (0.67, 1.72) | 1.26  (0.67, 2.33) | 0.92 | 1.24  (0.77, 1.99) | 1.46  (0.91, 2.33) | 0.94  (0.56, 1.54) | 0.98  (0.49, 1.88) | 0.58 |
| ***P* for trend** | 0.54 | 0.29 | 0.78 | 0.52 | - | 0.36 | 0.12 | 0.72 | 0.86 | - |
| **Alcohol drinking** | 0.85  (0.50, 1.41) | 0.72  (0.39, 1.28) | 0.86  (0.47, 1.54) | 1.10  (0.49, 2.40) | 0..89 | 0.84  (0.49, 1.42) | 0.66  (0.35, 1.19) | 0.95  (0.50, 1.78) | 1.14  (0.47, 2.67) | 0.77 |
| **Supplementary Table 5. Associations between the risk factors and MCI after stratified by age (adjusted ORs and 95 % CIs) (continued)** | | | | | | | | | | |
| **Variables** | **Sex adjusted** | | | | ***P* for heterogeneity**^c^ | **Multivariable adjusted** ^b^ | | | | ***P* for heterogeneity** |
|  | **60-64 (n=1,464)** | **65-69 (n=1,633)** | **70-74**  **(n=1,069)** | ≥**75**  **(n=465)** |  | **60-64 (n=1,464)** | **65-69 (n=1,633)** | **70-74**  **(n=1,069)** | ≥**75**  **(n=465)** |  |
| **Sleep duration (h)** |  |  |  |  |  |  |  |  |  |  |
| < 6.5 h | 0.91  (0.39, 1.83) | 1.29  (0.58, 2.55) | 0.63  (0.15, 1.83) | 0.97  (0.22, 3.15) | 0.78 | 0.91  (0.39, 1.89) | 1.29  (0.57, 2.60) | 0.57  (0.13, 1.69) | 0.88  (0.19, 3.02) | 0.72 |
| 6.5-8.5 h | 1.00 (reference) | 1.00 (reference) | 1.00 (reference) | 1.00 (reference) | - | 1.00 (reference) | 1.00 (reference) | 1.00 (reference) | 1.00 (reference) | - |
| > 8.5 h ^f, g, j, k^ | 1.07  (0.74, 1.54) | **1.67**  **(1.15, 2.42)** | **2.38**  **(1.60, 3.61)** | 1.41  (0.82, 2.47) | 0.41 | 0.94  (0.64, 1.37) | **1.69**  **(1.15, 2.48)** | **1.98**  **(1.31, 3.04)** | 1.26  (0.69, 2.33) | 0.43 |
| ***P* for trend** | 0.63 | 0.10 | **<0.0001** | 0.28 | - | 0.95 | 0.11 | **<0.01** | 0.45 | - |
| **Sleepiness (scores)** ^e, f, i, j^ | **2.78**  **(1.70, 4.53)** | **1.81**  **(1.07, 2.99)** | 1.36  (0.78, 2.33) | 1.40  (0.65, 2.92) | 0.21 | **2.66**  **(1.60, 4.39)** | **1.82**  **(1.07, 3.03)** | 1.40  (0.79, 2.46) | 1.08  (0.48, 2.37) | 0.20 |
| **BMI (kg/m^2^)** ^g, h, k, l^ | 0.99  (0.93, 1.04) | 1.00  (0.95, 1.06) | 0.90  (0.85, 0.96) | 0.83  (0.75, 0.91) | **<0.001** | 0.96  (0.90, 1.02) | 0.98  (0.92, 1.04) | **0.87**  **(0.81, 0.93)** | **0.82**  **(0.74, 0.90)** | **< 0.01** |
| **GS (per body weight) (kg/kg)** ^e, f, i, j, k, l^ | **0.48**  **(0.29, 0.81)** | **0.50**  **(0.30, 0.86)** | 0.61  (0.36, 1.06) | 0.55  (0.29, 1.07) | 0.95 | **0.51**  **(0.30, 0.89)** | **0.50**  **(0.28, 0.88)** | **0.51**  **(0.29, 0.93)** | **0.46**  **(0.23, 0.93)** | 1.00 |
| **Hypertension** ^g, k^ | 1.34  (0.95, 1.90) | 1.17  (0.82, 1.68) | **1.63**  **(1.10, 2.45)** | 1.39  (0.82, 2.40) | 0.67 | 1.32  (0.91, 1.91) | 1.12  (0.77, 1.64) | **1.79**  **(1.18, 2.78)** | 1.65  (0.93, 3.01) | 0.40 |
| **Diabetes** | 1.27  (0.81, 1.93) | 1.22  (0.78, 1.85) | 0.95  (0.58, 1.49) | 0.45  (0.17, 1.00) | 0.16 | 1.20  (0.75, 1.87) | 1.13  (0.71, 1.75) | 0.90  (0.54, 1.46) | 0.48  (0.17, 1.14) | 0.31 |
| **Hyperlipidemia** ^e, h^ | **0.66**  **(0.46, 0.94)** | 0.97 (0.67,1.43) | 0.76  (0.51, 1.13) | **0.57**  **(0.33, 0.97)** | 0.37 | 0.69 (0.47,1.00) | 0.99  (0.67, 1.47) | 0.83  (0.55, 1.27) | 0.83  (0.46, 1.49) | 0.62 |
| **TC (mmol/L)** ^j^ | 0.82  (0.38, 1.89) | 2.05  (0.88, 4.87) | 0.58  (0.25, 1.36) | 0.50  (0.15, 1.72) | 0.19 | 1.10  (0.48, 2.69) | **2.53**  **(1.05, 6.02)** | 0.70  (0.28, 1.79) | 0.71  (0.18, 2.79) | 0.20 |
| **TG (mmol/L)** | 0.72  (0.50, 1.02) | 0.95  (0.66, 1.38) | 0.68  (0.45, 1.00) | 0.63  (0.35, 1.12) | 0.61 | 0.73  (0.49, 1.07) | 0.88  (0.59, 1.31) | 0.76  (0.48, 1.18) | 1.18  (0.60, 2.29) | 0.63 |
| **LDL-C (mmol/L)** | 1.40  (0.73, 2.69) | 1.62  (0.84, 3.20) | 1.13  (0.55 ,2.36) | 1.01  (0.40, 2.65) | 0.91 | 1.64  (0.83, 3.27) | 1.74  (0.88, 3.49) | 1.19  (0.55, 2.59) | 1.26  (0.45, 3.59) | 0.87 |
| **HDL-C (mmol/L)** | 0.86  (0.39, 1.92) | 1.58  (0.72, 3.52) | 1.10  (0.52, 1.86) | 0.90  (0.30, 2.65) | 0.72 | 0.92  (0.39, 2.18) | 2.10  (0.89, 4.98) | 0.85  (0.33, 1.80) | 0.41  (0.11, 1.43) | 0.20 |
| BMI body mass index, GS grip strength, PA physical activity, METs metabolic equivalents, TG triglyceride;  ^a^ Obtained by using multiple logistic regression analysis.  ^b^ Additionally adjusted for education level, income status, marriage status, employment status, physical activity, smoking status, drinking status, sleep duration, BMI, GS, hypertension, diabetes, hyperlipidemia or its diagnostic indicators, sleepy state.  ^c^ Reflects comparison between odds ratio associated with man vs women using the statistical of heterogeneity.  ^d^ Physical activity (≥23.0 vs < 23.0).  ^e, i^ *P* <0.05 for sex adjusted and Multivariable adjusted in group of 60 to 64 years, respectively; ^f, j^ *P* <0.05 for group of 65 to 69 years; ^g, k^ *P* <0.05 for group of 70 to 74 years; ^h, l^ *P* <0.05 for group of aged 75 and over.  ^m^ unadjusted other variables.  ^n^ Boldface indicates statistical significance (*P*< 0.05) (or appropriate value). | | | | | | | | | | |
